# Enhancing iNKT cell immunotherapy through the integration of optimized CAR endodomains and iNKT engagers

**DOI:** 10.1038/s41467-026-76718-7

**Published:** 2026-08-14

**Authors:** Kanagaraju Ponnusamy, Klesti Karaxhuku, Yuchao Jiang, Lyra Randzavola, Hongwei Ren, Ilia Leontari, Bryan Lye, Mehmood Zaidi, Edward J. Bartlett, Edward W. Tate, Vasileios Pardalis, Dimitrios Leonardos, Reza Nadafi, Irene Sarkar, Rogier M. Reijmers, Marco Bua, Maria Atta, Alexia Katsarou, Irene AG Roberts, Aristeidis Chaidos, Anastasios Karadimitris

**Affiliations:** 1 Hugh & Josseline Langmuir Centre for Myeloma Research, Centre for Haematology, Department of Immunology and Inflammation, Imperial College London, London, UK; 2 Centre for Haematology, Department of Immunology and Inflammation, Imperial College London, London, UK; 3 Centre for Inflammatory Disease, Department of Immunology and Inflammation, Imperial College London, London, UK; 4 Department of Chemistry, Natural Sciences, Imperial College London, London, UK; 5 University of Ioannina Medical School, Ioannina, Greece; 6 Lumicks, Amsterdam, Netherlands; 7 Department of Haematology, Hammersmith Hospital, Imperial College Healthcare NHS Trust, London, UK; 8 Department of Paediatrics, University of Oxford, Oxford, UK; 9 Molecular Haematology Unit, MRC Weatherall Institute of Molecular Medicine, University of Oxford, Oxford, UK

**Keywords:** Myeloma, Cell therapies

## Abstract

iNKT cells are emerging as a highly promising immunotherapy platform for the treatment of cancer. To maximise the anti-cancer activity of CAR-iNKT against the blood cancer multiple myeloma we investigated optimal CAR designs and their combination with new iNKT-specific engagers. We find that amongst five different CAR endodomains, underpinned by increased avidity and a cross talk between Plexin D1 on CAR-iNKT and Semaphorin 4 A on myeloma cells, BCMA CD28z CAR-iNKT exert the highest anti-myeloma activity. Notably, CD28z CAR-iNKT outperform their CAR-T counterparts. To expand the anti-myeloma potential of CAR-iNKT, we designed and validated a high efficacy BCMA iNKT-specific engager which exerts significant anti-myeloma activity in conjunction with adoptively transferred iNKT cells. Finally, combined, dual target therapy with FCRL5 CAR-iNKT and BCMA iNKT engagers outperforms FCRL5 CAR-iNKT and limits immune escape of FCRL5-negative myeloma. Thus, optimised iNKT-based, dual-target, dual-modality immunotherapy has enhanced anti-tumor activity against multiple myeloma and potentially other malignancies.

## Abstract (web summary)

iNKT cells are a subset of T cells with both innate and adaptive features, emerging as a promising immunotherapy platform due to their rapid response. Here, the authors develop a CAR-iNKT strategy incorporating a CD28ζ co-stimulatory domain and two bispecific iNKT engagers to enhance multiple myeloma targeting.

## Introduction

Cellular immunotherapies, such as autologous chimeric antigen receptor (CAR)-T cells, have dramatically improved the outcome of patients with some blood cancers and are potentially curative for relapsed/refractory B non-Hodgkin lymphoma^1^ and B acute lymphoblastic leukemia^2^. Cure of the B lineage malignancy multiple myeloma (MM) has proved much more difficult to achieve. However, recent results using Cilta-cel, an autologous anti-BCMA CAR-T product, showed a 15% 5-year minimal residual disease-negative state and disease-free survival in multiple relapsed disease, suggesting curative potential for some patients^3^. We hypothesise that cure rates for MM could be increased by employing a strategy that would harness the properties of effectors more powerful than T cells, such as iNKT cells; optimise CARs, in particular their endodomain; and develop additional therapeutic modules, such as iNKT-specific engagers targeting additional myeloma-associated antigens to allow broader targeting of myeloma plasma cells.

Given their ability to protect from acute graft-versus-host disease (aGVHD)^4,5^, iNKT are emerging as an attractive allogeneic immune cell effector platform to autologous or allogeneic T cell-based products for the treatment of blood cancers and autoimmune diseases. iNKT, a rare subset of T cells, have features of both innate and adaptive immunity and possess effector as well as immunoregulatory activity. They are characterized by an invariant TCRVα24Jα18 pairing with a TCRVβ11 chain^6^, and are restricted by the glycolipid-presenting, MHC-like molecule CD1d^7^. Their anti-tumor activity is mediated by CD1d-dependent and -independent mechanisms, including direct killing of tumor cells, antigen-presenting cell (APC) maturation and activation of tumor-specific T cells^8,9^, and depletion of immunosuppressive, CD1d-expressing tumor-associated myeloid cells^10,11^. Moreover, iNKT can be deployed as a speedier, “off-the-shelf” immunotherapy platform without the need for deletion of the endogenous TCR and the additional genetic engineering this entails in allogeneic CAR-T cells, thereby reducing costs and manufacturing complexity. Additional potential advantages of allogeneic iNKT cell-over T cell-based immunotherapies include their ability to directly target CD1d-expressing tumors, such as myeloma plasma cells^12^, through the iTCR; the higher potency of CAR-iNKT over CAR-T as we and others have shown^13–17^, and a reduced risk for cytokine release syndrome and neurotoxicity^18^.

Efficacy of CARs is determined by several structural features, including the choice of the endodomain, such as CD28z vs 41BBz. CD28z CARs achieve swifter and more robust activation of T cells, while 41BBz CARs promote longer persistence of CAR-T but often fail to tackle CAR antigen-low disease^19^. Here, we describe a strategy to improve “off-the-shelf” cellular immunotherapy for MM by engineering CAR-iNKT cells equipped with an optimal CAR endodomain that includes the critical co-stimulatory domain(s). After comparing five co-stimulatory domains, we find that CD28z elicits the highest avidity against MM cells. Reflecting their enhanced avidity, CD28z CAR-iNKT outperform all other CAR-iNKT as well as CAR-T counterparts in in vivo models of MM. The increased avidity and in vivo anti-myeloma activity of CD28z CAR-iNKT is underpinned by a new mechanism that involves interaction of PLXND1 with SEMA4A, a PLXND1 ligand highly expressed on myeloma cells. To further enhance the therapeutic potential of CD28z CAR-iNKT, we developed a new iNKT cell-specific engagers, which, in combination with optimized CAR-iNKT, achieve broader myeloma antigen targeting and higher efficacy than single CAR-iNKT or iNKT plus bispecific engager and limit CAR target-negative disease.

## Results

### Proliferative and avidity advantage of CD28z CAR-iNKT

**Fig. 1:**
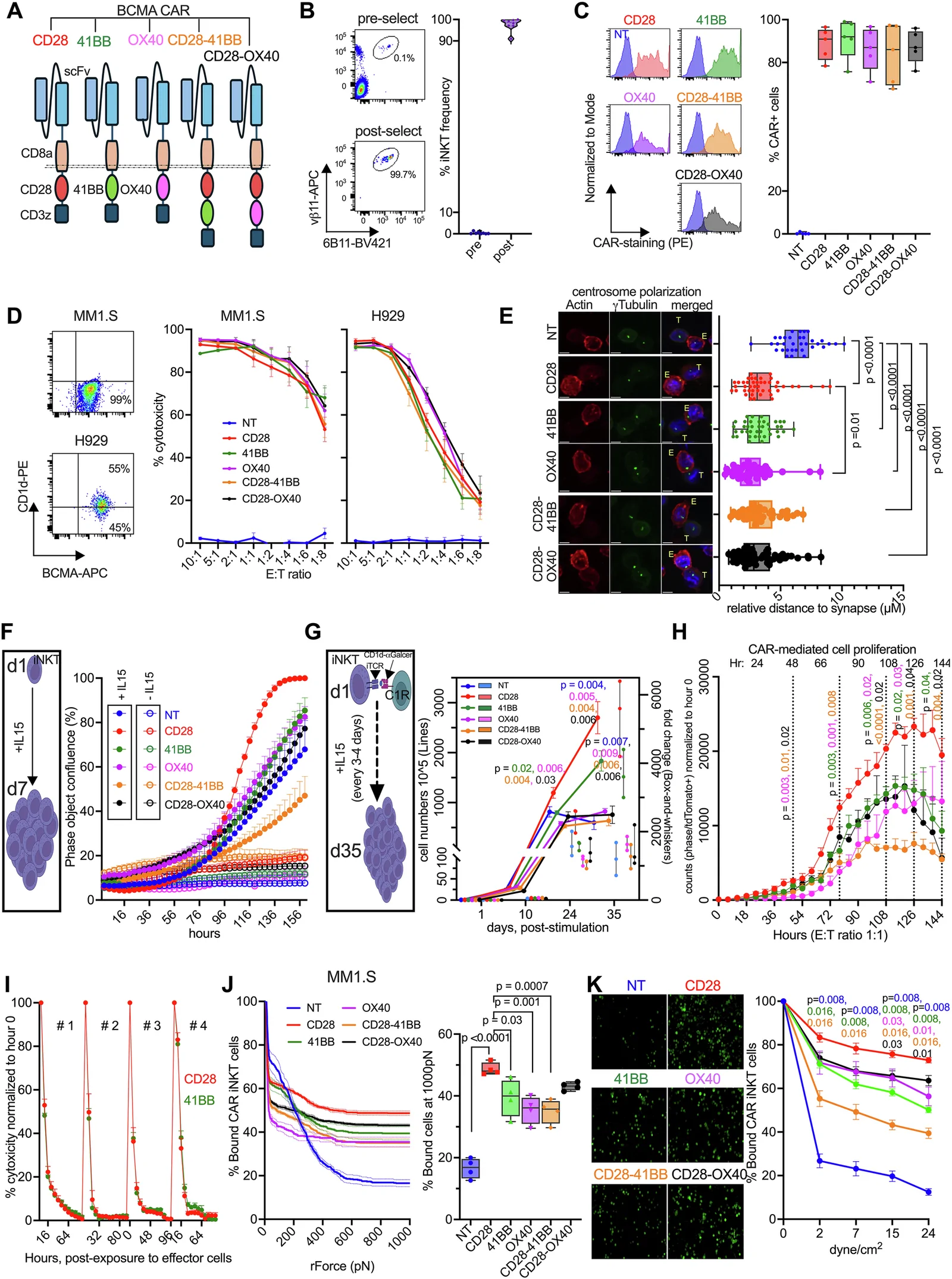
Higher proliferative potential and avidity of CD28z CAR-iNKT. **A** Structure of CARs. **B** Left: iNKT frequency in PBMC before and after selection as identified. Right: cumulative data (*n* = 8 healthy donors). **C** Transduction efficiency as assessed by flow cytometry (left) and cumulative data (right). **D** Surface expression of BCMA and CD1d detected by flow cytometry in MM1.S and H929 cells (left) and cytotoxic activity of BCMA CAR-iNKT cells as compared to non-transduced (NT) iNKT cells (*n* = 5 donors). **E** Immune synapse formation of BCMA CAR-iNKT cells conjugated to MM1.S target cells. 3D confocal microscopy reconstruction of z-stacks is shown and used to quantify the distance of the centrosome (green) to the synapse; distance <3 µM = polarised centrosome (*n* = 3 donors, each point represents a synapse point distance). **F** Increased short-term proliferative capacity of CD28z CAR-iNKT cells as compared to all other CAR- and NT- iNKT cells in the presence of IL-15 alone, or without external IL-15, assessed by real-time longitudinal Incucyte® imaging (*n* = 3 donors). *p* = 0.01, 0.0001, 0.047, <0.0001, 0.008 for NT, 41BBz, OX40z, CD28-41BBz, CD28-OX40z as compared to CD28z at 108 h. **G** Absolute numbers and fold expansion of CD28z CAR-iNKT after stimulation with alphaGalCer-loaded C1R-CD1d cells as compared to all other CAR-iNKT (*n* = 3 donors). **H** CD28z CAR-iNKT proliferation capacity as compared to other co-stimulatory domain CAR-iNKT in the presence of tdTomato-expressing MM1.S cells, without any cytokine supplementation (*n* = duplicates of 3 donors). Color code of the *p*-values matches the sample color as compared to CD28z. **I** Myeloma re-challenge assay. Cytotoxic activity of CD28z and 41BBz CAR-iNKT against MM1.S cells at E:T ratio 1:1 for four repeated challenges as assessed by real-time Incucyte® imaging (*n* = duplicates of 3 donors). **J** Left: CAR- and NT-iNKT cell binding capacity on MM1.S cells under increasing acoustic force; right: CAR-iNKT binding at rForce 1000 pN (representative of *n* = 2 donors, datapoints shown for individual runs). **K** Avidity of CAR-iNKT against MM1.S cells under shear stress in flow-chamber assay. Representative image of cell binding at 24 dyne/cm^2^, binding efficiency, and cumulative data. (*n* = 4 (OX40z, CD28-41BBz, CD28-OX40z) & 5 (NT, CD28z, 41BBz) donors). Two-tailed statistical analysis: Mann–Whitney *U* test (**E**, **K**), unpaired t test (**F**–**H**), and ordinary one-way ANOVA (**J**) were performed. All error bars show mean ± SEM; box-whiskers show minimum-to-maximum values. *p* < 0.0001 is referred as *p* < 0.0001. non significance measured by *p* > 0.05.

We generated five different lentiviral CARs against the established anti-myeloma target BCMA using the previously described and clinically validated anti-BCMA C11d5.3 scFv^20^, a G4S linker, CD8a-derived spacer, and transmembranous domains and either 2nd generation CD28z, 41BBz, OX40z or third generation CD28-41BBz and CD28-OX40z endodomains (Fig. 1A).

Using our previously published protocol, we then generated corresponding, same-donor highly pure CAR-iNKT with similarly high CAR expression levels (Fig. 1B, C).

Using different donors, we found that all five CAR-iNKT constructs were similarly cytotoxic against the BCMA high- and low-expressing H929 (CD1d+) and MM1.S (CD1d-) myeloma cells, respectively (Fig. 1D). As expected, reactivity was not observed against BCMA knock out H929 or BCMA-negative K562 cell lines (Supplementary Fig. 1A, B), confirming their specificity. Similarly, there were no significant differences in the production of granzyme B and interferon-γ (Supplementary Fig. 1C, D) and, although confocal microscopy imaging showed all CAR-iNKT established more pronounced immune synapses than non-transduced (NT) iNKT cells with myeloma cells as assessed by centrosome polarization at the immune synapse, again there were no differences between them (Fig. 1E).

However, short-term proliferation assays performed by real-time imaging (Fig. 1F) as well as assessment of long-term expansion potential in the presence of IL-15 and re-stimulation with CD1d-expressing B cell lines loaded with the glycolipid alpha-galactosylceramide (Fig. 1G), showed that CD28z CAR-iNKT expanded significantly more than CAR-iNKT with the other four endodomains, including 41BBz CAR-iNKT. In addition, 6-day Incucyte® S3 (Sartorius) real-time imaging-based, simultaneous assessment of effector proliferation and target cell cytotoxicity showed that CD28z CAR-iNKT cells display a significantly greater proliferative capacity in the presence of the BCMA-expressing MM1.S myeloma cells as compared to all other counterparts (Fig. 1H). By contrast, consistent with the 24 h cytotoxicity assays (Fig. 1D), there was no difference in the cytotoxic activity amongst the five CAR-iNKT effectors (Supplementary Fig. 1E).

A more detailed functional analysis focused on CD28z vs 41BBz CAR-iNKT showed that they had the same cytotoxic activity against an extended panel of myeloma cell targets (Supplementary Fig. 1F–H) independent of the CAR expression levels (Supplementary Fig. 1I, J). Further, in a tumor re-challenge assay, we found no significant differences in the cytotoxic activity between CD28z and 41BBz CAR-iNKT cells when repeatedly exposed to MM1.S target cells for four cycles (Fig. 1I); similarly, no significant differences in the expression of the exhaustion markers PD-1, LAG-3, and TIM-3 was observed (Supplementary Fig. 1K–M).

As in vivo potency of CAR-T cells is often better predicted by their avidity against their tumor targets^17,21–23^, we employed both acoustic force- and shear stress-based avidity assays (Fig. 1J, K) to study the strength of CAR-iNKT cell interaction with myeloma cells at steady state. We found that in both assays and using two different myeloma cells as targets, CD28z CAR-iNKT demonstrated the highest avidity amongst all five CAR-iNKT designs (Fig. 1J, K, and Supplementary Fig. 1N, O).

Therefore, higher proliferative potential and avidity distinguish CD28z from other 2nd and 3rd generation endodomains in the context of CAR-iNKT-mediated targeting of MM.

### PLXND1-SEMA4A interaction determines higher avidity of CD28z CAR-iNKT

**Fig. 2:**
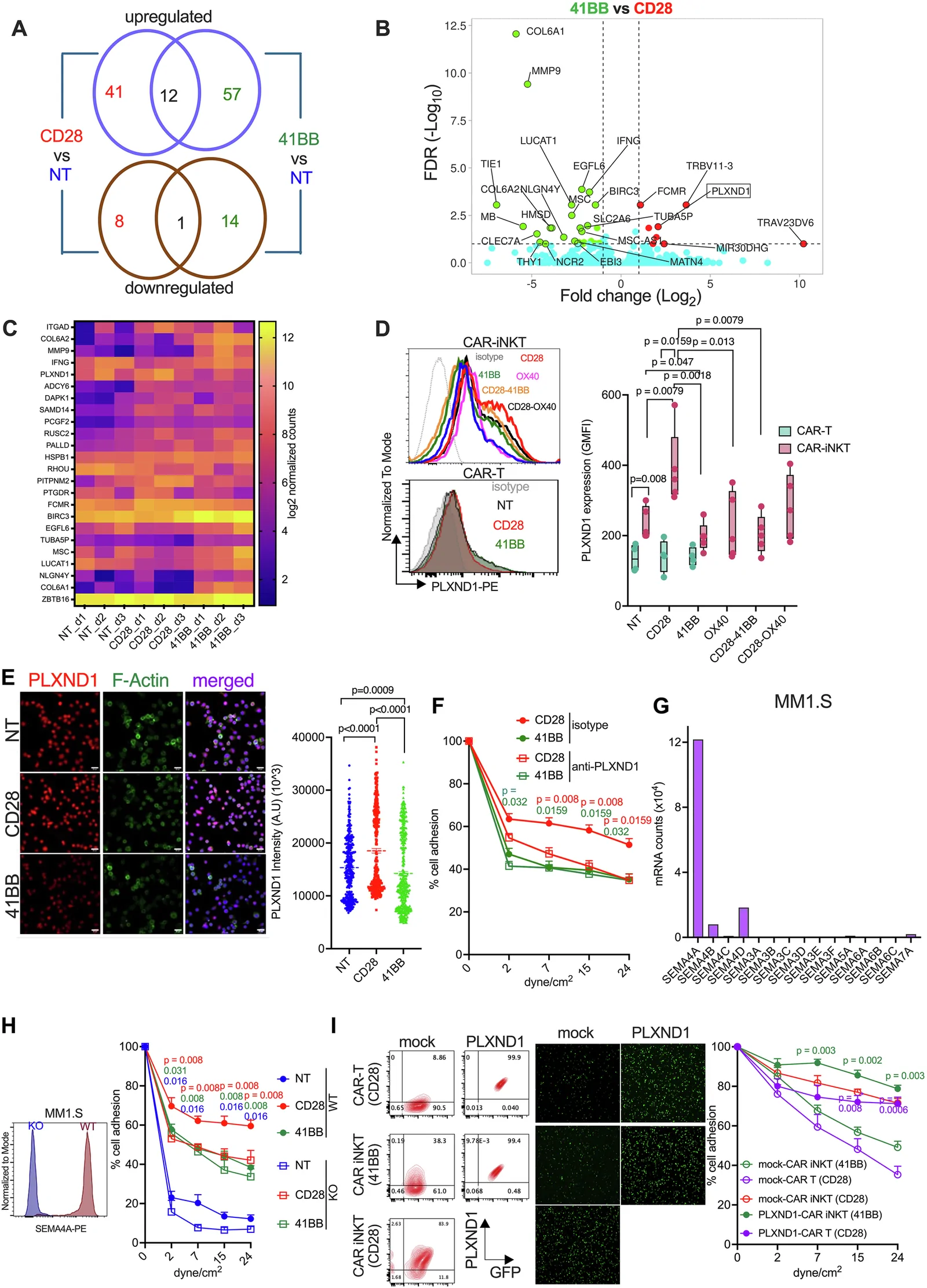
PLXND1-SEMA4A interaction determines higher avidity of CD28z CAR-iNKT. **A** Venn diagram shows the number of commonly shared differentially regulated genes between CD28z and 41BBz compared to NT iNKT cells at steady state (*n* = 3 donors (d1–d3)). **B** Volcano plot for differentially regulated genes between CD28z and 41BBz CAR iNKT cells at steady state (*n* = 3 donors). The names of the top 25 significantly regulated genes are shown, including PLXND1. **C** Heatmap shows the log2 count values of key genes differentially expressed in NT, CD28z, and 41BBz CAR iNKT cells. ZBTB16, a marker for iNKT cells shown as a control. (*n* = 3 donors). **D** PLXND1 expression in BCMA CAR iNKT cells with different co-stimulatory domains and NT iNKT as assessed by flow cytometry (histogram, top) and in BCMA CD28z and 41BBz CAR-T cells (histogram, bottom); right: geometric mean of fluorescence intensity (GMFI) cumulative data (*n* = iNKT (5 donors), T (4 donors). **E** Left: PLXND1 expression in CD28z CAR iNKT cells compared to NT and 41BBz iNKT cells as assessed by immunofluorescence; right: cumulative data of intensity of staining. Each dot represents a cell (*n* = 3 pooled donors). **F** Cell binding efficiency of CD28z CAR iNKT cells on MM1.S cells in the presence of PLXND1-blocking antibody or isotype control under shear stress (*n* = CD28z (5 donors) and 41BBz (4 donors). **G** Expression of *SEMA4A* mRNA and its family members in MM1.S cells from the CCLE dataset. **H** Left: SEMA4A expression in SEMA4A in wild type and knock-out MM1.S cells. Right: avidity of NT, CD28z and 41BBz CAR iNKT cells under shear stress against SEMA4A-expressing and non-expressing MM1.S cells (*n* = NT (4 donors), CD28z and 41BBz (5 donors each). **I** PLXND1 and GPF co-expression as assessed by flow cytometry of shown flow-sorted effectors on the basis of PLXND1 expression (left). Flow chamber avidity assay of indicated CAR iNKT cells over a range of shear stress levels (right) and representative images of indicated effectors bound to MM1.S myeloma cells at 24 dyne/cm^2^ (middle). (*n* = 3 donors). Two-tailed statistical analysis: Mann–Whitney *U* test (**D**–**F**, **H**), unpaired t-test (**I**) performed. All error bars show mean ± SEM, and box and whiskers show minimum to maximum values with all data points. *p* < 0.0001 is referred as *p* < 0.0001. non significance measured by *p* > 0.05.

Next, we investigated the mechanism that underpins higher avidity of CD28z CAR-iNKT. Since avidity is measured within minutes of adding resting effectors to their target cells, we reasoned that differences would be mediated by proteins already expressed on the surface of the different types of CAR-iNKT cells. To identify such proteins, we compared the transcriptomes by RNA-sequencing of resting CD28z and 41BBz CAR-iNKT as well as NT NKT from three different donors. We found a small number of differentially expressed genes between CD28z or 41BBz CAR-iNKT vs iNKT cells and 28z vs 41BBz CAR-iNKT (Fig. 2A–C and Supplementary Dataset 1). Both CD28z and 41BBz CAR-iNKT upregulate key genes related to cell adhesion and migration, such as *ITGAD*, *MMP9*, *COL6A2,* and also *IFNG* (Fig. 2C). Amongst the few differentially expressed genes, we found that *PLXND1*, a semaphorin ligand family receptor, was significantly upregulated in CD28z CAR-iNKT (Fig. 2B, C). Flow cytometry analysis confirmed significantly higher levels of expression of PLXND1 in CD28z CAR-iNKT as compared to 41BBz, other CAR-iNKT designs and NT iNKT (Fig. 2D), a finding also corroborated by quantitative immunofluorescence (Fig. 2E). Expression of PLXND1 remained stable and did not increase upon CAR-iNKT activation in the presence of myeloma cells (Supplementary Fig. 2A) while no significant differences of PLXND1 expression between CD4+ and CD4- iNKT cells were observed (Supplementary Fig. 2B). In contrast to CAR-iNKT, PLXND1 was not detected on CD28z or 41BBz CAR-T cells by flow-cytometric analysis (Fig. 2D) although published single cell RNA-seq data shows that while naïve T cells lack or display very low expression of PLXND1, memory and particularly TEMRA CD4+ and CD8+ subsets express higher levels (Supplementary Fig. 2C, D)^24,25^.

To further explore the role of PLXND1, we repeated the shear stress avidity assays in the presence of a blocking PLXND1 mAb^26^. In relation to the Ig isotype control, this resulted in a dose-dependent reduction of CD28z CAR-iNKT avidity but had no effect on the avidity status of 41BBz counterparts (Fig. 2F and Supplementary Fig. 2E). Therefore, the higher avidity of CD28z CAR-iNKT over NT and 41BBz CAR-iNKT is PLXND1-dependent.

To investigate this further, we measured gene expression of *SEMA4A* and *SEMA3E*, the semaphorin family members which could potentially act as ligands for PLXND1^27–29^, in the two myeloma cell lines used in the avidity assays. While expression of *SEMA4A* was the highest amongst all semaphorins, *SEMA3E* was not expressed in either MM1.S or H929 cells (Fig. 2G and Supplementary Fig. 2F), with the same expression pattern seen in primary myeloma plasma cells from patients with MM (Supplementary Fig. 2G). Of note, SEMA4A has been validated as a therapeutic target for CAR- and Ab-based immunotherapy of MM, and similar to FCRL5^30,31^, another clinically validated bispecific engager target, its expression is highest in the high-risk chr1q-amp myeloma cells^32^ (Supplementary Fig. 2H).

To confirm the role of SEMA4A in CAR-iNKT avidity, using CRISPR/Cas9 gene editing, we generated MM1.S myeloma cells lacking expression of SEMA4A (Fig. 2H). Although the cytotoxic activity of CD28z and 41BBz CAR-iNKT was the same against both SEMA4A-expressing and gene-edited SEMA4A-negative MM1.S cells (Supplementary Fig. 2I), avidity of CD28z CAR-iNKT against SEMA4A-negative myeloma cells was lower compared to SEMA4A-expressing myeloma cells, and at the same level as that of the 41BBz CAR-iNKT cells which was unaffected by abolishing SEMA4A expression (Fig. 2H). These findings demonstrate that enhanced avidity of CD28z CAR-iNKT requires expression of SEMA4A on myeloma cells.

Furthermore, we tested whether differential cell proliferation and avidity induced by the CD28z co-stimulatory domain would be independent of the CAR target. For this purpose, we synthesised 41BBz and CD28z CARs against FCRL5. The scFv of these CARs was based on the clinically active FCRL5 engager cevostomab^32^ and was previously validated in the context of CAR-T therapy^33^. We found that, as for BCMA CAR-iNKT, CD28z and 41BBz FCRL5 CAR-iNKT cells show similar cytotoxicity against FCRL5-expressing MM1.S myeloma cells (Fig. 6A and Supplementary Fig. 2J). However, CD28z FCRL5 CAR iNKT cells showed higher proliferative potential than 41BBz FCRL5 CAR iNKT or NT iNKT cells (Supplementary Fig. 2K) and higher expression of PLXND1 than their 41BBz counterparts, commensurate with higher PLXND1-dependent avidity against FCRL5+ MM1.S cells (Supplementary Fig. 2L).

Finally, we cloned the *PLXND1* cDNA into a lentiviral vector and exogenously over-expressed it in BCMA CAR-T and CAR-iNKT cells, and the transduced CAR-iNKT and CAR-T cells were flow-sorted to purity based on GFP/PLXND1 (Fig. 2I) followed by assessment of their avidity on MM1.S cells under shear stress (Fig. 2I and Supplementary Fig. 2M). We found that overexpression of PLXND1 increased the avidity BCMA 28z CAR-T cells and BCMA 41BBz CAR iNKT cells to the levels of BCMA CD28z CAR-iNKT cells. These data further corroborate the notion that higher PLXND1 expression underpins the higher avidity of CD28z CAR-iNKT and eventually their superior anti-myeloma activity when compared to 41BBz CAR-iNKT.

Together, these findings demonstrate that the higher avidity of CD28z CAR-iNKT is mediated by its higher expression of PLXND1 and its ability to engage SEMA4A on myeloma cells, and it is independent of the CAR scFv and its myeloma target.

### CD28z CAR-iNKT exert a more effective anti-myeloma activity in vivo in a PLXND1-dependent manner

**Fig. 3:**
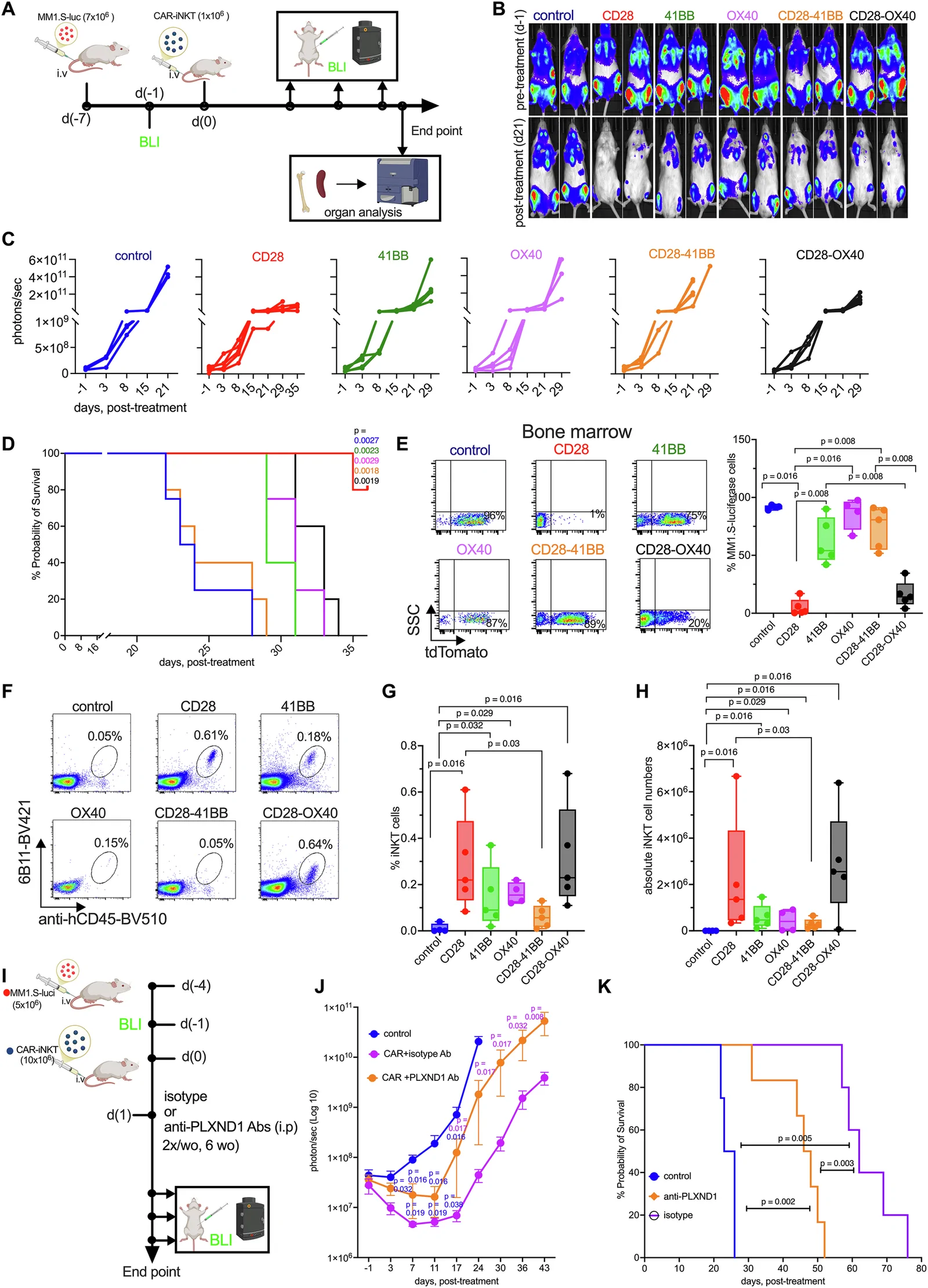
CD28z CAR-iNKT outperform other CAR designs in a PLXND1-dependent manner. **A** Schematic of in vivo experimental design associated with (**A**–**H**). **B**, **C** Representative BLI images of mice from control and BCMA CAR iNKT with different co-stimulatory molecules-treated groups for before treatment (day −1) and post-treatment (day 21), and its cumulative photon strength shown for each group. (*n* = 4 mice (control and OX40z each group), 5 mice (CD28z, 41BBz, CD28-41BBz, and CD28-OX40z each group). **D** Kaplan–Meier survival curve for all the groups treated with 1 × 10^6^ BCMA CAR iNKT cells and the control group. **E** Left: representative flow cytometry dot plots and its cumulative data (right) for frequency of MM1.S-luci cells as measured by tdTomato fluorescence marker by flow cytometry in the bone marrow of all the groups collected at end point. (*n* = 4 mice (control and OX40z each group), 5 mice (CD28z, 41BBz, CD28-41BBz, and CD28-OX40z each group). Representative flow cytometry analysis plots for iNKT frequency in the bone marrow for each group (**F**), and its cumulative data (**G**). (*n* = 4 mice (control and OX40z each group), 5 mice (CD28z, 41BBz, CD28-41BBz, and CD28-OX40z each group). **H** Absolute iNKT cell numbers in the bone marrow of each group were counted based on flow cytometry analysis using counting beads. (*n* = 4 mice (control and OX40z each group), 5 mice (CD28z, 41BBz, CD28-41BBz, and CD28-OX40z each group). **I** Schematic of in vivo experimental design associated with (**J**, **K**). **J** Total body BLI signals of mice treated with anti-Plexin D1 antibody-treated group as compared to isotype antibody-treated group and untreated control group (**J**). **K** Kaplan–Meier survival curve for (**J**). (*n* = 4 mice (control group); 5 mice (isotype group); and 6 mice (Ab group). Two-tailed statistical analysis: Mann–Whitney *U* test (**E**, **G**, **H**, **J**) and ordinary one-way ANOVA (N). The Gehan–Breslow–Wilcoxon test (**D**, **K**) was performed. All error bars show mean ± SEM, and box and whiskers show minimum to maximum values with all data points. *p* < 0.0001 is referred as *p* < 0.0001. non significance measured by *p* > 0.05.

To investigate whether avidity indeed predicts anti-myeloma activity in vivo, we employed a systemic model of MM using luciferase- and tdTomato-expressing MM1.S (MM1.S-Luc) myeloma cells and monitored tumor burden by bioluminescence imaging (BLI). To better delineate differences between CAR constructs we used the limiting dose^34^ of 10^6^ CAR-iNKT cells per animal for each group (Fig. 3A). We found that CD28z CAR-iNKT followed by CD28-OX40z CAR-iNKT were the most potent in delaying tumor growth amongst the five CAR types as assessed by BLI (Fig. 3B–D and Supplementary Fig. 3A, B) while prolongation of survival was more pronounced in animals treated with CD28z CAR iNKT (Fig. 3D).

Immunophenotypic analysis of bone marrow, spleen and peripheral blood confirmed significantly lower myeloma burden in CD28z CAR iNKT-treated mice (Fig. 3E and Supplementary Fig. 3C, D). The CAR-iNKT frequency and absolute numbers in the bone marrow at end point, although did not reach statistical significance, were higher in CD28z and CD28-OX40z CAR-iNKT compared to other CAR-iNKT groups (Fig. 3F–H). Together, these data validate the avidity assay prediction that CD28z CAR-iNKT are the most potent amongst the five CAR-iNKT groups.

Finally, we investigated the impact of PLXND1 blockade in vivo by treating myeloma-bearing mice with CD28z CAR-iNKT cells plus either PLXND1 blocking Ab or isotype (Ig) control as shown in (Fig. 3I). While both groups of CAR iNKT were effective in controlling disease progression as compared to untreated controls, survival of CAR-iNKT plus PLXND1 Ab-treated animals was significantly shorter than that of CAR-iNKT plus Ig-treated animals (Fig. 3J, K, and Supplementary Fig. 3E).

Therefore, we conclude that PLXND1-dependent higher avidity accounts for the higher anti-myeloma activity of CD28z CAR-iNKT in vivo.

### CAR-iNKT outperform CAR-T against myeloma

**Fig. 4:**
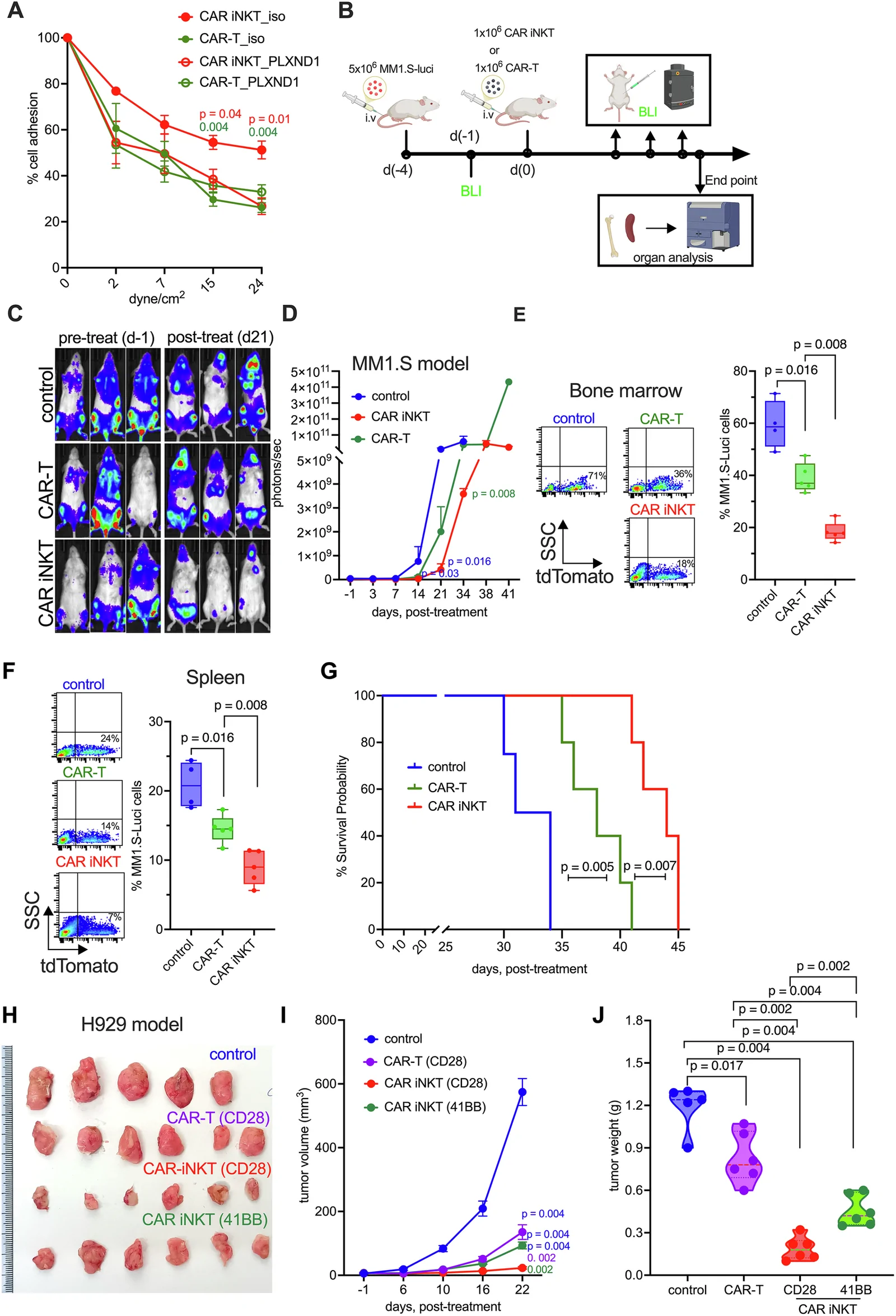
CAR iNKT outperform CAR-T against myeloma. **A** BCMA CAR iNKT cell avidity on MM1.S cells shown as compared to BCMA CAR-T cells of the same donors with isotype or PLXND1 blocking antibody at 1 µg/ml under shear stress (*n* = 3 donors). *p*-value color matches the compared sample color. **B** Schematic of in vivo experimental design associated with (**A**–**G**). Representative BLI images of mice from the control, BCMA CAR iNKT and BCMA CAR-T cell-treated group before (day −1) and after treatment (day 21) (**C**) and its cumulative photon emission shown for each group (**D**). (*n* = 4 mice (control group); 5 mice (treatment group each). Left: representative dot plots and cumulative data (right) for frequency of MM1.S-luci cells in the bone marrow (**E**) and spleen (**F**) analysed by flow cytometry using the tdTomato fluorescence marker at end point. (*n* = 4 mice (control group); 5 mice (treatment group each). **G** Kaplan–Meier survival curve for myeloma-bearing mice treated with CAR iNKT or CAR-T as compared to untreated control as shown in (**B**). (*n* = 4 mice (control group); 5 mice (treatment group each). **H** Images of CD1d+ H929 subcutaneous myeloma tumor retrieved from NSG mice treated with 1 × 10^6^ indicated effector cells. **I** Volume of subcutaneous myeloma tumor over time in NSG mice treated with indicated effectors. (*n* = 5 mice (control group); 6 mice (treatment group each). **J** Weight of above subcutaneous myeloma tumors at end of experiment. (*n* = 5 mice (control group); 6 mice (treatment group each). Two-tailed statistical analysis: unpaired t-test (**A**), Mann–Whitney *U* test (**D**–**F**, **I**, **J**). The Gehan–Breslow–Wilcoxon test (**G**) was performed. All error bars show mean ± SEM, and box and whiskers show minimum to maximum values with all data points. *p* < 0.0001 is referred as *p* < 0.0001. non significance measured by *p* > 0.05.

Next, since our previous work demonstrated that iNKT cells are a more powerful immunotherapy platform for CAR therapy of lymphoma and high-risk ALL^13,17^, we investigated whether this is also the case in MM. Indeed, CD28z BCMA CAR-iNKT from three different donors were consistently more cytotoxic towards MM1.S myeloma cells than their CAR-T counterparts (Supplementary Fig. 4A) and exhibited higher PLXND1-dependent avidity in vitro than CAR-T (Fig. 4A). In line with these observations when used at the limiting dose of 10^6^ CAR+ effectors (Fig. 4B), CAR-iNKT were significantly more potent than CAR-T counterparts in vivo in delaying growth of systemic myeloma (Fig. 4C, D, and Supplementary Fig. 4B, C) and depleting myeloma cells from bone marrow and spleen (Fig. 4E, F). Accordingly, survival of CAR-iNKT-treated mice was significantly longer than that of CAR-T counterparts (Fig. 4G). Finally, in a subcutaneous model of myeloma involving the CD1d+ H929 myeloma cells, CD28z BCMA CAR-iNKT administered at the limiting dose of 10^6^ CAR+ cells/mouse were more effective than CAR-T counterparts in delaying tumor growth as assessed by tumor *volume* in vivo and tumor weight at endpoint (Fig. 4H–J and Supplementary Fig. 4D). Importantly CD28z CAR-iNKT were also more effective than 41BBz CAR-iNKT, thus further corroborating the findings from the systemic myeloma model (Fig. 3D). Of note, we did not observe any clinical toxicity in either systemic or sbc myeloma models of CAR-iNKT immunotherapy. Reflecting this, there were no differences in the weight of treated animals as compared to untreated controls (Supplementary Fig. 4C, D).

Together, these data show that CD28z BCMA CAR-iNKT outperform CAR-T counterparts against myeloma, likely in a PLXND1-dependent manner.

### Development, specificity, and anti-myeloma activity of iNKT cell engagers

Having identified the optimal CAR endodomain, we hypothesised that CAR-iNKT-based immunotherapy could be further enhanced by developing iNKT cell-specific engagers that would target additional myeloma-associated antigens. Such engagers would bring CAR-iNKT cells to the proximity of tumor cells, facilitating their killing, as previously described for T and NK cell-specific bi- and tri-specific engagers in pre-clinical/clinical development or already licensed for clinical use^35^.

To provide proof-of-concept in support of this hypothesis, we designed two bispecific iNKT engagers (biNTe). In principle, an iNKT engager could be designed to engage either the TCRVβ11 or TCRVα24 variable, non-CDR3 segments of the iTCR or the TCRVα24Jα18 CDR3 idiotype. Here, we designed two engagers against TCRVβ11, based on the TCRVβ11 Ab clone C21^6^. Since engagement of iTCR with CD1d and lipid ligands involves primarily the TCRVα24Jα18 CDR3, such an approach would be expected to allow iNKT activation through iTCR-CD1d interaction even in the presence of a TCRVβ11 engager.

The tumor targeting arm of the engagers was designed to bind to BCMA, and like the CAR, it was based on the C11d5.3 scFv^20^ with modifications in the linker.

**Fig. 5:**
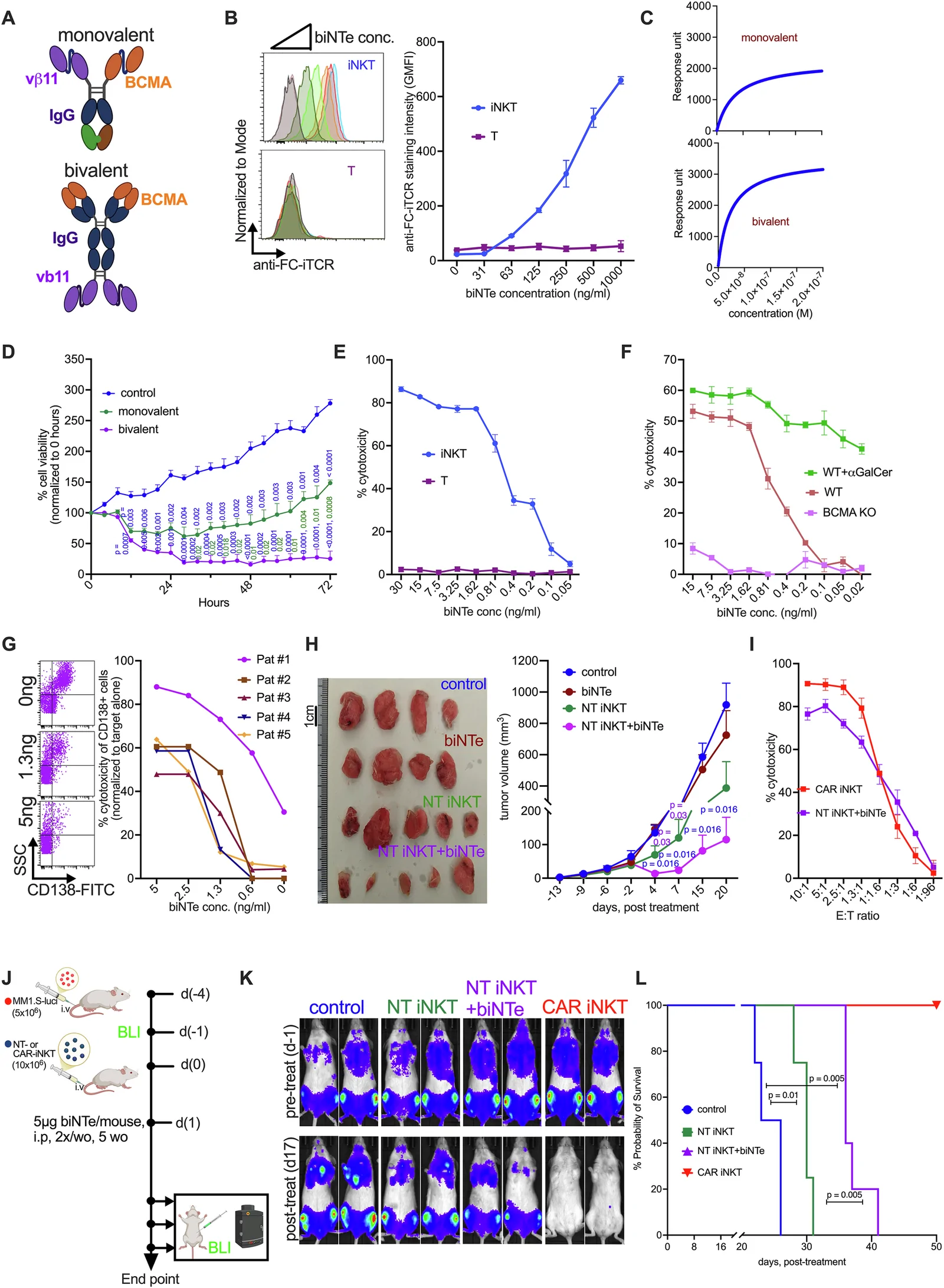
Development and anti-myeloma activity of iNKT- specific engagers. **A** Design of monovalent and bivalent biNTe. **B** Left: flow-cytometric analysis of same-donor purified iNKT and T cells after staining with increasing concentrations (0–1 µg) of bivalent biNTe; (right) cumulative data is shown as GMFI values for iNKT and T cells (*n* = 3 donors). **C** Representative data showing affinity to soluble BCMA of bivalent vs monovalent biNTe as determined by surface plasmon resonance (SPR) (*n* = 3 donors). **D** Cytotoxic activity of NT-iNKT cells incubated with bivalent vs monovalent biNTe against MM1.S as assessed by real-time live Incucyte® imaging for 72 h (*n* = 3 donors). **E** Cytotoxic activity of same-donor iNKT and T cells incubated with bivalent biNTe against MM1.S myeloma cells (representative *n* = 3 donors). **F** Cytotoxic activity of iNKT incubated with biNTe in the presence or not of alphaGalCer against BCMA-expressing parental and BCMA knock out CD1d+ H929 myeloma cells (*n* = 3 donors). **G** iNKT-biNTe-mediated cytotoxicity of patient-derived primary bone marrow myeloma cells at 2:1 E:T ratio and different biNTe antibody concentrations for 20 h. Left: representative dot plot for patient #1. Right: cumulative data normalized to target alone (*n* = 5 patients). CD138+ cells were measured for each condition and normalized to myeloma target cells alone. **H** MM1.S cell subcutaneous tumor size (left) and volume (right) treated with NT-iNKT and/or biNTe and untreated mice. (*n* = 4 mice (control and biNTe group each), 5 mice (iNKT and iNKT+biNTe group each). *p-* value color codes matches the compared sample line. **I** Cytotoxic activity of NT-iNKT cells incubated with biNTe and BCMA CAR-iNKT cells against MM1.S cells shown for 16 h (*n* = 3 donors). **J** Schematic of experimental design shown for MM systemic model for biNTe treatment related to (**K**, **L**). **K** Left: representative BLI of mice with systemic myeloma treated with BCMA CAR-iNKT or NT iNKT plus biNTe before (day −1) and after treatment (day 17); cumulative data shown in Supplementary Fig. 5K (*n* = 4 mice (control and NT iNKT group each), 5 mice (iNKT+biNTe and CAR iNKT group each). **L** Kaplan–Meier survival of myeloma-bearing mice treated as shown in (**J**). Two-tailed statistical analysis: unpaired t-test (**D**), Mann–Whitney *U* test (**H**), and the Gehan–Breslow–Wilcoxon test (**L**) were performed. All error bars show mean ± SEM. *p* < 0.0001 is referred as *p* < 0.0001. non significance measured by *p* > 0.05.

One biNTe design comprised a heterodimeric, monovalent, scFv-Fc human wild-type IgG1 with Fc-silent (L234A/L235A (LALA) and a “key-in-hole” changes (Fig. 5A). A second biNTe design comprised a homodimeric, bivalent IgG-scFv human wild-type IgG1. The anti-BCMA arm has a Fab configuration while the anti-TCRVb11 arm has a scFv configuration (Fig. 5A).

After cloning of the constructs into an expression vector and their transfection to 293T cells, protein was purified from the culture supernatant using Protein A columns, and biNTe purity was confirmed by SDS-PAGE (Supplementary Fig. 5A).

Using the biNTe to stain purified iNKT and T cells followed by fluorescent-labelled secondary anti-human IgG1 Fc Ab, we found that compared to controls, biNTe-stained purified iNKT but not T cells in a dose-dependent manner (Fig. 5B and Supplementary Fig. 5B), thus demonstrating the specific binding of the iNKT cell arm of biNTe to the iNKT TCRVβ11 chain.

We next confirmed the ability of the tumor-binding arm of biNTe to bind their intended target, BCMA, showing that staining of iNKT cells occurred only in the presence of bio-sBCMA (Supplementary Fig. 5C). To assess the potency of binding, we employed surface plasmon resonance. This showed a lower Kd and thus higher affinity for bivalent over monovalent biNTe to soluble BCMA (Kd of 23.4 nM vs 34.5 nM respectively) (Fig. 5C and Supplementary Fig. 5D–F). In line with these findings, EC50 measured against MM1.S cells in the presence of iNKT was lower for bivalent than monovalent biNTe (2.4 pM vs 6.2 pM respectively) (Supplementary Fig. 5G, H). Similarly, the bivalent biNTe demonstrated significantly higher longitudinal cytotoxic potential in vitro compared to the monovalent engager (Fig. 5D). Based on its higher affinity and cytotoxic potential, the bivalent engager was selected for further study.

Bivalent biNTe selectively killed myeloma cells in the presence of iNKT, but not T cells supporting the specificity of the biNTe iTCR arm (Fig. 5E). In addition, the anti-myeloma cytotoxic activity of iNKT against CD1d-expressing BCMA+ (but not BCMA-) H929 myeloma cells were further enhanced in the presence of biNTe and aGalCer (Fig. 5F) confirming the BCMA specificity of the tumor engaging arm of the biNTe and BCMA- and iNKT cell-dependent killing of myeloma cells. These findings also validate the prediction that binding of the designed biNTe to the TCRVβ11 chain would allow the iTCR to engage with CD1d on tumor cells and to further enhance anti-myeloma activity.

We next co-cultured bone marrow cells from five patients with myeloma with iNKT cells and varying concentrations of biNTe. We found that while the frequency of myeloma PC dramatically decreased in a biNTe dose-dependent manner, that of the non-myeloma bone marrow cells was considerably less affected, suggesting specific killing of the myeloma but not of the non-myeloma bone marrow cells (Fig. 5G and Supplementary Fig. 5I).

To corroborate the in vitro findings, we tested the in vivo efficacy of the biNTe in a subcutaneous myeloma model using the BCMA-expressing MM1.S cells. After tumor engraftment, mice were left untreated or treated with either 10^7^ unmodified, pre-expanded iNKT and 5 μg/mouse biNTe ip 24 h post-iNKT treatment and then twice a week (Fig. 5H and Supplementary Fig. 5J). While iNKT or biNTe alone had no impact on tumor growth, the iNKT and biNTe combination significantly delayed myeloma tumor growth, first observed 2 days after the first engager dose (Fig. 5H), thus providing proof-of-principle that biNTe, when combined with adoptive iNKT immunotherapy, has significant anti-myeloma activity.

We next compared the anti-myeloma activity of BCMA biNTe with that of BCMA CAR-iNKT cells. Myeloma MM1.S cells were co-cultured with NT iNKT and biNTe or BCMA CAR-iNKT at a 1:1 E:T ratio. Cytotoxicity against MM1.S cells showed slightly lower killing by biNTe plus iNKT compared to CAR-iNKT, attesting to the potency of the BCMA biNTe (Fig. 5I). Finally, we compared the efficacy of BCMA biNTe plus iNKT vs BCMA CAR-iNKT in a systemic myeloma model using 10^7^ effectors (Fig. 5J). We found that although CAR-iNKT were overall more efficacious in vivo, the BCMA biNTe plus iNKT combination also significantly delayed disease progression as assessed by BLI, reduced myeloma cell frequency in bone marrow, and increased overall survival as compared to iNKT-only-treated and untreated controls (Fig. 5K, L, and Supplementary Fig. 5K–M).

Finally, there were no differences in the longitudinally measured weight of biNTe-treated animals (Supplementary Fig. 5J, M), suggesting a lack of significant toxicity.

We conclude that biNTe are specific for iNKT and in combination with adoptive transfer of in vitro expanded iNKT, exert a considerable anti-myeloma activity.

### biNTe and CAR-iNKT for dual targeting of multiple myeloma

Having established an optimal CAR endodomain for CAR-iNKT treatment of myeloma and proof-of-principle of the anti-myeloma activity of biNTe, we next tested whether combining the two therapeutic modules would enhance the anti-myeloma activity of the iNKT cell platform by targeting two different antigens simultaneously, i.e., the clinically validated targets BCMA and FCRL5 by bivalent biNTe and 28z CAR, respectively.

**Fig. 6:**
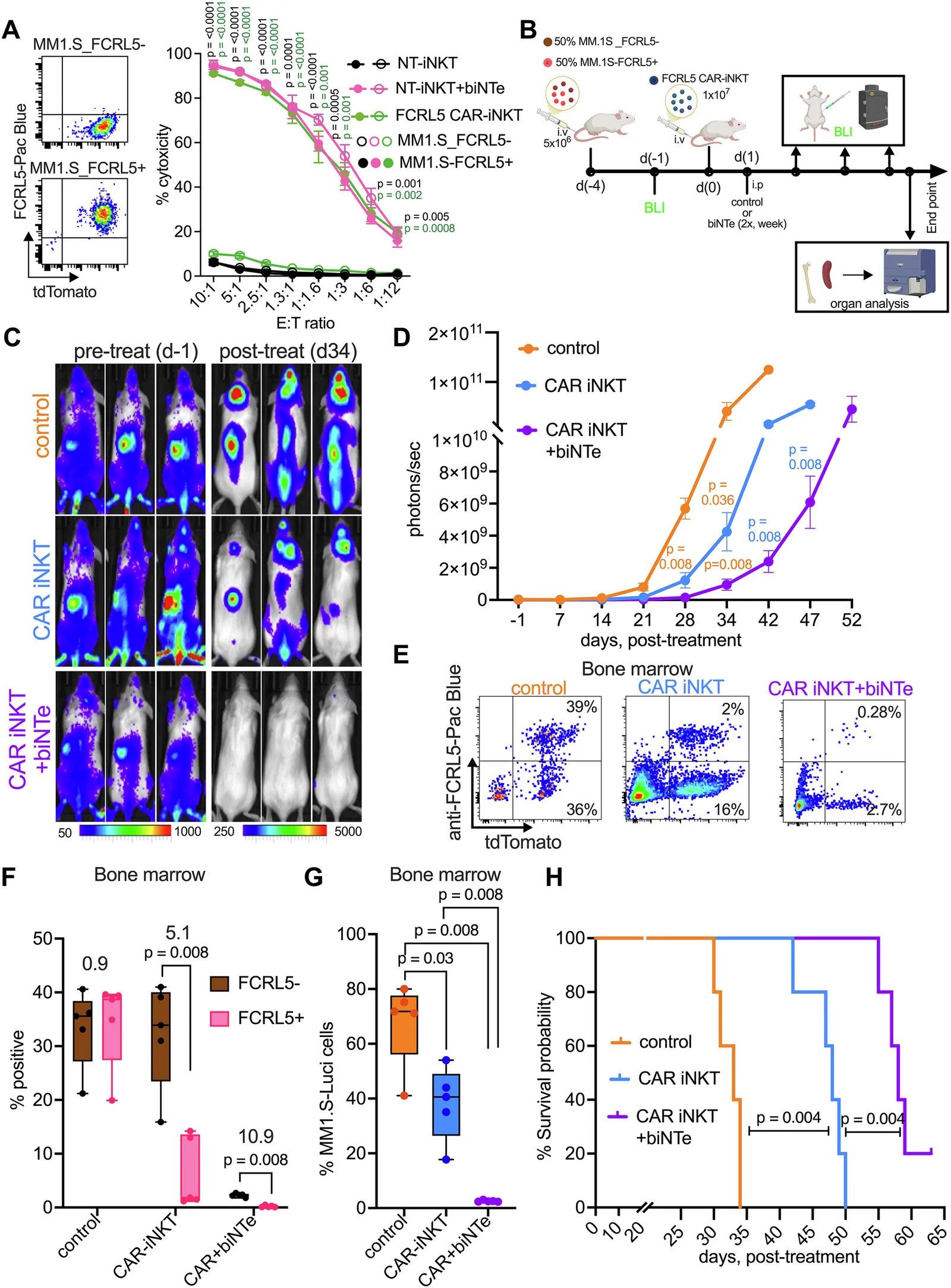
Enhanced anti-myeloma activity by dual CAR-iNKT plus biNTe targeting. **A** Flow cytometry plots show exogenous expression of FCRL5 in MM1.S (MM1.S-FCRL5+) cells and corresponding WT (MM1.S_FCRL5-) cells (left) and cytotoxic activity of FCRL5 CAR iNKT or NT iNKT cells incubated with or without biNTe against MM1.S_FCRL5- and MM1.S-FCRL5+ cells (right). (*n* = 3). **B** Schematic of in vivo experimental design associated with Fig. 6. Representative BLI images of mice from the control and FCRL5 CAR-iNKT with or without biNTe group before (day −1) and after treatment (day 28) (**C**) and their cumulative BLI data (**D**). (*n* = 5 mice per group). Significance color code indicates corresponding sample color. Flow cytometry analysis at end point of the frequency of bone marrow MM1.S_FCRL5- and MM1.S-FCRL5+ cells in treated and control mice shown as a dot plot (**E**) and cumulative data for each group (**F**). Numbers indicate the ratio between FCRL5- and FCRL5+ cells. (*n* = 5 mice per group). **G** Flow cytometry analysis at end point of total MM1.S myeloma burden as determined by tdTomato fluorescence marker in the bone marrow of treated and control mice. (*n* = 5 mice per group). **H** Kaplan–Meier survival curve of myeloma-bearing mice left untreated (control) or treated with FCRL5 CAR iNKT cells or FCRL5 CAR iNKT plus biNTe (*n* = 5 mice per group) as shown in (**B**). Two-tailed statistical analysis: unpaired t-test (**A**), Mann–Whitney *U* test (**D**, **F**, **G**), and the Gehan–Breslow–Wilcoxon test (**H**) were performed. All error bars show mean ± SEM, and box and whiskers show minimum to maximum values with all data points. *p* < 0.0001 is referred as *p* < 0.0001. non significance measured by *p* > 0.05.

For this purpose, we first established that FCRL5 CD28z CAR iNKT cells are cytotoxic against FCRL5+ but not FCRL5- MM1.S myeloma cells (Fig. 6A). We then used a systemic myeloma model generated by a 50–50 mixture of BCMA+FCRL5+ and BCMA+FCRL5- MM1.S-Luc cells, i.e., BCMA and FCRL5 are expressed by 100% and 50% respectively of tumor cells (Fig. 6B). Such a set up would test the ability of biNTe to enhance the anti-myeloma activity of the CAR and limit immune escape even when half of the tumor cells lack expression of the CAR target.

These myeloma-bearing mice were left untreated or were treated with 10^7^ FCRL5 CAR-iNKT or FCRL5 CAR-iNKT plus BCMA biNTe. The combination of FCRL5 CAR-iNKT plus BCMA biNTe significantly prolonged disease progression to a greater extent than CAR-iNKT (Fig. 6C, D). Assessment of the fraction of FCRL5- and FCRL5+ myeloma cells in bone marrow and spleen at end-point confirmed the expected ratio of ~1:1 in untreated animals while in FCRL5 CAR-iNKT plus BCMA biNTe and CAR-iNKT-treated animals this ratio was significantly higher at 10.1:1 and 5.1:1 respectively in favor of FCRL5- cells (Fig. 6E, F, and Supplementary Fig. 6A), consistent with a strong selective effect of FCRL5 CAR-iNKT against FCRL5+ disease. The overall disease burden in bone marrow and spleen was also significantly lower in CAR-iNKT plus BCMA biNTe compared to CAR-iNKT-treated animals (Fig. 6G and Supplementary Fig. 6B), consistent with the ability of biNTe to enhance the anti-myeloma activity of the CAR even when half of the tumor cells lack expression of the CAR target. In line with this, overall survival of FCRL5 CAR-iNKT plus BCMA biNTe-treated animals was significantly longer than that of animals treated with CAR-iNKT (Fig. 6H). Again, compared to untreated animals, no differences in the weight of treated animals were observed (Supplementary Fig. 6C).

Together, these data provide strong proof-of-principle evidence that the combined CAR-iNKT plus biNTe modality immunotherapy enhances the therapeutic potency of the iNKT cell platform and can limit immune escape of tumor cells lacking the CAR target antigen in myeloma.

## Discussion

As iNKT cells emerge as a potentially more advantageous allogeneic immunotherapy platform than autologous or allogeneic T cells for cancer, there is a need to maximize their efficacy by optimising the therapeutic modules with which they are engineered. This would be particularly pertinent to multiple myeloma, an incurable blood cancer for which conventional autologous anti-BCMA CAR-T therapy may have potential to induce durable complete remissions in a small minority of patients^3^.

Here, we describe a strategy to improve INKT-based “off-the-shelf” cellular immunotherapy for MM first by optimising CAR design. CAR design, especially CD28z vs 41BBz endodomain choice, impacts efficacy and persistence of CAR-T cells by differential activation of downstream signalling pathways^19^. While CD28z supports robust T cell activation and more efficient targeting of CAR antigen-low disease in part due to a lower level of trogocytosis by CAR-T cells of CAR antigen^36^ and robust phosphorylation of CD3z by LCK^37^, 41BBz promotes longer-term persistence of CAR-T cells, in part through activation of the non-canonical NFkB pathway^38^. Here, our systematic and multilayered comparison of five 2nd and 3rd generation CAR endodomain designs provides robust evidence that in the context of CAR-iNKT immunotherapy for myeloma, a CD28z design would be the most advantageous in terms of potency without evidence of reduced persistence. Our data shows that a new mechanism involving PLXND1-SEMA4A interaction determines differential avidity and thus higher anti-myeloma activity of CD28z CAR-iNKT as compared to CAR-iNKT with four different CAR endodomain designs. While engagement of PLXND1 on endothelial cells by SEMA4A has been shown to limit angiogenesis^29^, little is known on the role of PLXND1-SEMA4A in modulating the function of immune cells. Plxnd1, through its interaction with Sema3E, is required for migration of positively selected thymocytes into the medulla of the thymus^39^, while interaction of the Plxnd1-associated receptor Neuropilin 1 with Sema4A promotes transcriptional and functional stability of Tregs^40^. Of the potential PLXND1 ligands^27^, only SEMA4A is expressed in myeloma cells, hence its validation as a target for Ab- and CAR-based immunotherapy of multiple myeloma^30^. SEMA4A is also expressed in several other cancers and is required for checkpoint inhibitor-induced enhancement of anti-tumor T cell responses in lung cancer^41^, while Sema4A-expressing dendritic cells enhance activation and differentiation of antigen-specific T cells via engagement of Tim2^42^. Additional work will be required to determine the signalling pathways that operate downstream of PLXND1 and underpin this advantage of CD28z CAR-iNKT.

Since both *FCRL5* and *SEMA4A* are chr1q genes and are overexpressed in myeloma cells with chr1q-amp, a critical adverse prognosis genetic feature, CD28z-based CAR-iNKT approach targeting FCRL5 in combination with BCMA could be particularly advantageous for this high-risk myeloma^43^. This contrasts with the fact that both clinically licensed anti-BCMA CAR-T products for MM feature a 41BBz endodomain. However, since pre-clinical comparison of CD28z vs 41BBz FCRL5 CAR-T demonstrated the superior ability of the former to control myeloma in vivo^31^, whether 41BBz would be the preferred endodomain for CAR-T treatment of MM remains an open question. Our data also highlight the need for testing and identifying optimal CAR co-stimulatory domains for each innate lymphocyte subset such as NK and γδT cells.

In terms of potency, consistent with previous work by us and others showing higher anti-tumor activity of CAR-iNKT in pre-clinical B cell lymphoma, high-risk B ALL, and solid tumors^13–17^, we demonstrate that CAR-iNKT also outperforms CAR-T in the context of multiple myeloma. This advantage of iNKT over T cells could be mediated both directly by the iTCR interacting with CD1d expressed on some tumors, including myeloma cells^12^, and engagement of activating NK receptors (e.g., NKG2D) by their ligands expressed by tumor cells; or indirectly- through enhancement of innate and adaptive anti-tumor immune responses, as well as targeting and depletion of CD1d-expressing immunosuppressive and tumor-promoting myeloid cells^10,15,16,44^. The latter mechanism, although not directly addressed in this work, would also be expected to contribute to the anti-tumor activity of CAR-iNKT cells in multiple myeloma given that tumor-associated myeloid cells have been shown to suppress effective anti-myeloma T cell responses and promote myeloma cell survival^45^.

Guided by pre-clinical and recent early clinical work demonstrating the anti-cancer potential of bispecific engagers administered in combination with adoptive transfer of effectors, such as NK cells^46^, and to expand the options for multi-modular targeting of multiple myeloma, we developed iNKT cell-specific engagers and demonstrated their anti-myeloma activity. Such engagers, with the exception of a γδTCR engager that cross-reacts with the TCRVα24Jα18 CDR3 idiotype of the iTCR^47^, have not been described. Our TCRVβ11-engaging bispecific antibody has two distinct properties: firstly, the option to target any tumor-associated antigen with one arm; and secondly, the availability of the TCRVα24Jα18 CDR3 loop to engage with CD1d-presented glycolipids, thus further contributing to CD1d-dependent tumor killing. Between the two bispecific engager designs, i.e., monovalent vs bivalent, the latter appears to be more potent in in vitro assays, likely reflecting its higher affinity and when tested against soluble BCMA and higher avidity when tested against myeloma cells in the presence of iNKT cells. We envisage that iNKT engagers, with single or dual anti-tumor specificity, can be used flexibly in conjunction with other therapeutic modalities, such as single or dual CAR and/or TCR, thus achieving dual, triple, or even quadruple targeting of myeloma, other blood or solid tumor cancers. Clinical development of this modular therapy would entail infusions of the iNKT cell engagers in conjunction with transfer of autologous or allogeneic, in vitro expanded iNKT or CAR/TCR-iNKT cells.

In summary, in the context of multiple myeloma, we provide proof-of-concept that iNKT engineered with optimally selected CAR designs display superior anti-myeloma activity over CAR-T cells, and their efficacy can be further broadened and enhanced by addition of iNKT cell engagers.

## Methods

### Ethics statement

The peripheral blood of unidentifiable healthy donor was received from NHS Blood and Transplant, UK, under ethical approval (NCI no: 2758). Primary myeloma cells were collected under ethical committee approval (REC no. 11/H0308/9) from bone marrow samples derived from myeloma patients. For tumor models in animals, the study was conducted by following the licence (PPL no. PP2944557) provided by the UK Home Office. The maximum tumor size exceeding 12.5 mm diameter or weight loss >15% or hunching, piloerection lasting >24 h or tumor inflammation with ulceration or signs of extensive bone marrow involvement, such as reduced limb movement, or when tumors obstruct normal movement set as humane end points. The tumor burden was measured by vernier calliper for subcutaneous or bioluminescence imaging for systemic models, and the maximal tumor burden was not exceeded and the experiments were terminated upon reaching the humane endpoints or scientific objectives.

### iNKT isolation and expansion, cell culture, primary cells

iNKT cells were isolated from healthy donor-derived peripheral blood mononuclear cells (PBMCs) using anti-iNKT microbeads (cat no. 130-094-842; Miltenyi Biotec) followed by stimulation with irradiated PBMCs and anti-CD3 and anti-CD28 antibodies and cultured in R10 media (RPMI 1640 supplemented with 10% fetal bovine serum and 1% Pen/strep, 4 mM L-glutamine, 1% non-essential amino acids, 1 mM sodium pyruvate, 15 mM Hepes buffer, 0.05 mM 2-mercaptoethanol) supplemented with 150 U IL-15 (cat. no 130-095-764; Miltenyi Biotec) and expanded as described^13,17^. The myeloma cell lines MM1.S cells (CRL-2974, ATCC) and NCI-H929 cells (CRL-3580, ATCC) were cultured in RPMI 1640 supplemented with 10% fetal bovine serum and 1% Pen/strep, 1% L-glutamine, 1% non-essential amino acids, and 1% sodium pyruvate. The myeloma cells were identified by staining the cells with anti-human CD138 (Syndecan-1) (clone: MI15) and anti-human CD38 (clone: HIT2).

### Cloning, lentiviral transduction

BCMA CAR sequences were modified from Carpenter et al.^23^ and modified by inserting a (G4S)3 linker between the light and heavy chain of scFv and appropriate costimulatory molecules and CD8a hinge and transmembrane and 7AA of cytoplasmic domain as shown in Fig. 1A, and cloned into a second-generation plasmid containing SFFV promoter and WPRE. CAR lentiviral particles were packaged in 293T cells by transfection of 4.1 µg CAR plasmid with packaging plasmids 5.4 µg psPAX2 and 2.9 µg pMD2.G plasmid for 48 h. The viral particles were concentrated by ultracentrifugation at 23,000 RPM, 4 °C. The cells were transduced on 8 µg/ml retronectin-coated plates by inoculating viral particles and cells at 1000 × *g* at 37 °C.

### CD28 BCMA CAR AA sequences

MALPVTALLLPLALLLHAARPDIVLTQSPPSLAMSLGKRATISCRASESVTILGSHLIHWYQQKPGQPPTLLIQLASNVQTGVPARFSGSGSRTDFTLTIDPVEEDDVAVYYCLQSRTIPRTFGGGTKLEIKGGGGSGGGGSGGGGSQIQLVQSGPELKKPGETVKISCKASGYTFTDYSINWVKRAPGKGLKWMGWINTETREPAYAYDFRGRFAFSLETSASTAYLQINNLKYEDTATYFCALDYSYAMDYWGQGTSVTVSSFVPVFLPAKPTTTPAPRPPTPAPTIASQPLSLRPEACRPAAGGAVHTRGLDFACDIYIWAPLAGTCGVLLLSLVITLYCNHRNRSKRSRLLHSDYMNMTPRRPGPTRKHYQPYAPPRDFAAYRSRVKFSRSADAPAYQQGQNQLYNELNLGRREEYDVLDKRRGRDPEMGGKPRRKNPQEGLYNELQKDKMAEAYSEIGMKGERRRGKGHDGLYQGLSTATKDTYDALHMQALPPR* (CD28 domain sequences)

**41BB domain:** KRGRKKLLYIFKQPFMRPVQTTQEEDGCSCRFPEEEEGGCEL

**OX40 domain:** ALYLLRRDQRLPPDAHKPPGGGSFRTPIQEEQADAHSTLAKI

### CD28-41BB domain

RSKRSRLLHSDYMNMTPRRPGPTRKHYQPYAPPRDFAAYRSKRGRKKLLYIFKQPFMRPVQTTQEEDGCSCRFPEEEEGGCEL

### CD28-OX40 domain

RSKRSRLLHSDYMNMTPRRPGPTRKHYQPYAPPRDFAAYRSALYLLRRDQRLPPDAHKPPGGGSFRTPIQEEQADAHSTLAKI

These costimulatory domains were replaced in the CD28 BCMA CAR to create all the BCMA CARs with different costimulatory molecules.

### FCRL5 CAR AA sequences

MALPVTALLLPLALLLHAARPTGEVQLVESGPGLVKPSETLSLTCTVSGFSLTRFGVHWVRQPPGKGLEWLGVIWRGGSTDYNAAFVSRLTISKDNSKNQVSLKLSSVTAADTAVYYCSNHYYGSSDYALDNGQGTLVTVSSGGGGSGGGGSGGGGSDIQMTQSPSSLSASVGDRVTITCKASQDVRNLVVWFQQKPGKAPKLLIYSGSYRYSGVPSRFSGSGSGTDFTLTISSLQPEDFATYYCQQHYSPPYTFGQGTKVEIKFVPVFLPAKP TTTPAPRPPTPAPTIASQPLSLRPEACRPAAGGAVHTRGLDFACDIYIWAPLAGTCGVLLLSLVITLYCNHRNRSKRSRLLHSDYMNMTPRRPGPTRKHYQPYAPPRDFAAYRSRVKFSRSADAPAYQQGQNQLYNELNLGRREEYDVLDKRRGRDPEMGGKPRRKNPQEGLYNELQKDKMAEAYSEIGMKGERRRGKGHDGLYQGLSTATKDTYDALHMQALPPR* (CD28 domain sequences)

### Flow cytometry and cytotoxicity assay

iNKT cells were stained with anti-human CD3 (clone: UCHT1), anti-human TCR Valpha24-Jalpha18 (iNKT cell) (clone: 6B11), anti-human TCR Vbeta11 (clone: REA559) anti-human CD45 (HI30), anti-human CD8 (clone: SK1) and anti-human CD4 (clone: OKT4) for 30 min at 4 °C in FACS buffer (1× PBS + 0.5% HSA). The cells were stained with 7AAD after washing and analysed using flow cytometry. PlexinD1 (cat no. FAB4160P; R&D Systems), anti-human CD307e (FCRL5) (clone: REA391), and anti-human BCMA (clone: REA315) antibodies were used. CAR functional tests were performed by co-culturing CAR iNKT cells with target cells expressing CAR antigen at different E:T ratio in R10 media. The target cells were stained with cell trace violet (cat. no C34557; ThermoFisher), and the effector cells were depleted of cytokines overnight. After the indicated time of co-culture, the cells were washed with FACS buffer and cell viability was determined by staining the cells with 7AAD and flow cytometry. For primary myeloma cells, the total target cells were stained with cell trace violet before the coculture with NT iNKT cells, and the cells were stained with anti-CD138 antibodies to determine the frequency of myeloma plasma cells after 20 h of culture and analysed using flow cytometry. The data were normalized to target cells alone control. The MM1.S cells were incubated with recombinant BCMA protein (clone: 10620-H40H-B-SIB) to block BCMA in the biNTe experiment, followed by incubation with iNKT cells (1:1) and biNTes at EC50 dose. For iNKT staining, the cells were stained with CD3 APC-Cy (clone: UCHT1), CD4 PE (clone: OKT4), CD8 FITC (clone: RPA-T8), anti- TCR Va24-Ja18 BV421 (clone: 6B11), anti-TCR Vb11-APC (Order no. 130-108-733).

### Intracellular cytokine measurement

Non-transduced (NT) iNKT and BCMA CAR iNKT cells were co-cultured with MM1.S cells in the presence of 1× Brefeldin A overnight in R10 media without any cytokine stimulation. The cells were fixed and permeabilized by following standard manufacturer’s instructions (BioLegend, cat# 420801 and 421002). The cells were stained with granzyme B (clone: GB11) and IFNγ (clone: 4S.B3) in FACS buffer for 30 min, 4 °C and measured in flow cytometry after washing twice with FACS buffer.

### Avidity assay

The iNKT cell avidity was determined using z-Movi ® Cell Avidity Analyzer (Lumicks/Lumicks CA BV). Briefly, 10^6^ cells/ml BCMA-expressing MM1.S or H929 cell monolayer was prepared on poly-L-lysine-coated temperature-controlled microfluidic chips (Lumicks/Lumicks CA BV) for 1 h. Simultaneously NT or CAR-modified iNKT cells were stained with CellTrace Far Red dye (cat no. C34564; ThermoFisher) by following the manufacturer’s instructions and incubated on the monolayer for 5 min. The indicated acoustic force was applied, and the fraction of bound cells was measured. For shear stress models, cell avidity was measured by applying shear stress where MM1.S cell monolayer was prepared on 1 µg/ml retronectin-coated ibid-treated slides for 1 h at 37 °C. Simultaneously, the effector iNKT cells were stained with Cell Trace Green (cat no. C2925; ThermoFisher) or far-red dyes. The iNKT cells were incubated on the monolayer for 5 min and the indicated shear stress was applied continuously with an interval of 30 s between incremental shear stress levels. The total number of stained iNKT cells in the field was captured under fluorescence microscopy, and the images were captured at every shear stress level. For PlexinD1 blocking, iNKT cells were incubated with either 1 µg/ml isotype or anti-PLXND1 neutralizing antibody (cat no. PA5-47012; ThermoFisher) for 1 h, 37 °C and washed them in R10 media before applying them on the monolayer to measure the cell avidity efficiency as stated above. SEMA4A knock-out cells were generated by electroporating MM1.S cells with SEMA4A CRISPR gRNA and guide (156156422 CUCCAACAGGGGAUGAACGU-Modified) and SpCas9 (Synthego). The bound cells at all shear stress levels were counted using ImageJ software and normalized to bound cells at pre-shear stress application (0 dyne/cm^2^). For the PLXND1 overexpression model, PLXND1 full cDNA was cloned into LeGO-iG2 (Addgene #27341), which express EGFP as a fluorescence marker, and transduced into expanded BCMA CAR iNKT cells and BCMA CAR-T cells through lentiviral transduction followed by FACS purification for pure PLXDN1 expression cells. The avidity status of mock (i.e., EGFP only)-transduced or PLXDN1 overexpressed cells were studied using a shear stress flow model as stated above.

### Cell proliferation and tumor challenge in real-time Incucyte® S3 imaging

5 × 10^3^ CAR iNKT cells with different co-stimulatory molecules were incubated with MM1.S cells, which express tdTomato as a fluorescence marker, at 1:1 E:T ratio without IL15 cytokine supplement. The cytotoxicity potency was monitored by tdTomato fluorescence levels and normalized to hour 0. The cell proliferation of CAR iNKT cells after stimulation with target cells were calculated by total live cells (phase object) to tdTomato+ (Red fluorescence object) target MM1.S cells and normalized to hour 0 in real-time live imaging using Incucyte® S3 software. For the tumor challenge assay, 1 × 10^4^ BCMA CAR iNKT cells and target cells, tdTomato+ MM1.S cells, were incubated at 1:1 E:T ratio for 4 days without cytokine IL15 and repeated for four challenges by adding target cells at 1:1 E:T ratio. TdTomato fluorescence signals were measured in real-time live imaging using the Incucyte® S3 system. The exhaustion markers were measured in flow cytometry using PD-1 (clone: A17188A), LAG-3 (clone: 11C3C65), and TIM-3 (clone: 7D3) antibodies levels before the challenge and after the 4th challenge.

### Systemic and subcutaneous in vivo models of myeloma and bioluminescence

MM1.S cells were transduced with retrovirus to express the luciferase gene and tdTomato, an enhanced, tandem-dimeric, monomeric red fluorescent protein derived from the original DsRed protein as a fluorescence marker (MM1.S-Luci). To establish a systemic myeloma model, 5–7 × 10^6^ MM1.S-luci cells were injected intravenously to engraft in NOD SCID Gamma (NSG) mice (8–12 weeks old). The animals were purchased from Charles River, UK, and caged in individually ventilated cages, social housing with enriched bedding, with sterile food and water, and maintained at 20–24 ˚ C, 45–65% humidity, 12 h light cycle. The mice were imaged for bioluminescent signal (BLI) to track the disease burden under 2% isoflurane (Zoetis UK)/medical oxygen administration via inhalation and one single dose injection of 150 mg/Kg D-Luciferin injection, potassium salt (CAS No. 115144-35-9; Merck) in PBS via the intraperitoneal (IP) route for 10 min before imaging the mice. The images were captured for up to 3 min at auto mode and medium binning of IVIS for both the ventral and dorsal sides of each mouse. The region of interest (ROI) data analysis was performed using Living Image software and expressed in radiance (unit of photons/s/cm^2^/sr), and the total BLI was calculated by adding both dorsal and ventral side signals. For the subcutaneous model, 10^7^ MM1.S myeloma cells were injected with 200 µl Matrigel® Basement Membrane Matrix (cat no. 354234; Corning) subcutaneously, and the tumor size was measured using a vernier calliper, and tumor volumes were assessed using the formula (Volume = 1/2 × Length × Width^2^). To establish the H929 cells subcutaneous model, 5 × 10^6^ H929 cells were subcutaneously injected with 200 µl Matrigel® Basement Membrane Matrix. The tumor volume was measured as stated above. For treatment, NT iNKT or CAR-iNKT cells or CAR-T cells were injected through the tail vein intravenous route. For biNTe treatment, 5 µg biNTe antibody per mouse was injected intraperitoneally twice a week for up to 5 weeks. For PLXND1 blocking in vivo, the BCMA CAR iNKT-treated group mice were injected with either 5 µg goat anti-human isotype or PLXND1 neutralizing antibody per mouse (cat no. PA5-47012; ThermoFisher) intraperitoneally for 6 weeks, twice a week. The untreated and isotype control of the BCMA-treated group shown in Fig. 3K were served as negative and positive control, respectively, shown in Fig. 5L, as these experiments were performed at the same period and were designed to reduce animal usage and to comply with the 3R’s policy. Upon reaching humane end points of MM disease, the mice were sacrificed, and the organs were collected, followed by cell preparation in FACS buffer and measured for tdTomato to track MM1.S cells in flow cytometry.

### RNA sequencing

iNKT cells were transduced with BCMA CARs and expanded as stated above after purification of BCMA CAR iNKT cells on day 5 post-transduction, to have >95% CARs for all the BCMA CARs, by staining the cells with recombinant BCMA-biotin (cat no. 10620-H40H-B-SIB; Stratech Scientific) followed by magnetically labelled anti-streptavidin PE beads-based purification by MACS magnetic column. The CAR and iNKT purity was assessed by flow cytometry before isolation of the total RNA, and RNA sequencing was performed after enriching for poly-A mRNA and sequenced by Novogene.

### Surface plasmon resonance (SPR)

Kinetics data were collected on Biacore S200 Biosensors (Cytiva). Soluble biotinylated BCMA were immobilised to Series S SA sensorchips, which were maintained with a continuous flow of PBS-P+ (20 mM phosphate buffer with 2.7 mM KCl, 137 mM NaCl, and 0.05% Surfactant P20). SA sensorchips were prepared as per Cytiva protocols; the sensor surface was conditioned with three consecutive 1-min injections of 1 M NaCl in 50 mM NaOH and washed with 70% isopropanol in 1 M NaCl and 50 mM NaOH after each ligand injection. The biotin-tagged ligand was injected at 20 ng/µL for 1800 s, reaching an RU of 3500. Kinetic data were collected via SCK experiments with a 4-point dilution series (0.1, 1, 10, and 200 nM) in PBS-P+. The diluted compound series was injected in order of ascending concentration for 400 s, across a specific flow cell. Dissociation was monitored during a 400 s injection of assay buffer. An equivalent series of injections was carried out using Assay Buffer, which was used to correct flowcell-specific artefacts. Kinetics and affinity data were gathered using the Biacore Evaluation software.

### Bispecific antibody development

Monovalent biNTe composed of heterodimeric iTCR (clone: C21) scFv and anti-BCMA (clone: C11D5.3) scFv linked with Fc human wild-type IgG1 with Fc-silent (L234A/L235A (LALA) and a “key-in-hole” changes. A homodimeric IgG–scFv bispecific bivalent was constructed as the heavy chain (HC) encoded an anti-BCMA (C11D5.3) IgG (VH–CH1–hinge–CH2–CH3) C-terminally fused to an anti-Vβ11 scFv (C21) via a (G₄S)₃ linker. The light chain (LC) encoded the matched anti-BCMA (C11D5.3) VK–CK. HC and LC were cloned in separate mammalian expression vectors. The biNTes were expressed by transiently co-transfecting 30 µg of both plasmids of dimeric biNTe with 180 µg PEI (cat no. 7854; biotechne) (PEI:DNA 3:1 w/w mixed in 2 ml plain DMEM media) in 293T cells at 85% confluence grown in a T175 flask. The media was replaced with 30 ml fresh media after 24 h and added additional 30 ml fresh media replaced with 30 mL fresh complete DMEM (10% FBS, penicillin/streptomycin, glutamine) after 24 h and added additional 30 mL complete medium after 48 h. Cultures were maintained at 37 °C, 5% CO_2_. On day 6, culture supernatants were collected and clarified by two sequential centrifugations. The clarified supernatant was then filtered through a 0.4 µm membrane. The engagers were purified using rProtein A/Protein G GraviTrap column (cat no. GE28-9852-56; Cytiva) by passing 60 ml of culture supernatant, followed by washing twice with 10 ml 1× PBS. The antibody was eluted in 1 ml fractions of 0.1 M glycine-HCL (pH 2.7) elution buffer and neutralized by 60 µl 1 M Trizma hydrochloride solution, pH 9.0 (cat no. T2819; Merck), and the elution was repeated for six times. The eluted biNTe were concentrated using Amicon Ultra-15 centrifugal filters (Diameter (Metric): 29.7 mm MWCO: 30,000 d) (cat no. UFC903024; Merck) and washed twice with 1× PBS. The purity of biNTe was assessed by SDS-PAGE under reducing and non-reducing conditions.

### iTCR scFv AA sequences

DIKMTQSPSSMYASLGERVTITCKASQDINSYLSWFQQKAGKSPKTLIYRANRLVDGVPSRFSGSGSGQDYSLTISSLEYEDMGIYYCLQYDEFPFTFGGGTRLEIKGGGGSGGGGSGGGGSQVQLQQSGPEVVRPGVSVKISCKGSGYRFTDSAMHWVKQSHAKSLEWIGVISSYNGNTNYNQKFKGKATMTVDKSSSTAYMELARMTSEDSAIYYCARSRDAMDYWGQGTSVTVSS

### Anti-BCMA scFV AA sequences

DIVLTQSPPSLAMSLGKRATISCRASESVTILGSHLIHWYQQKPGQPPTLLIQLASNVQTGVPARFSGSGSRTDFTLTIDPVEEDDVAVYYCLQSRTIPRTFGGGTKLEIKGGGGSGGGGSGGGGSQIQLVQSGPELKKPGETVKISCKASGYTFTDYSINWVKRAPGKGLKWMGWINTETREPAYAYDFRGRFAFSLETSASTAYLQINNLKYEDTATYFCALDYSYAMDYWGQGTSVTVSS

### Monovalent (anti-BCMA scFV-IgG) AA sequences

MPLLLLLPLLWAGALADIVLTQSPPSLAMSLGKRATISCRASESVTILGSHLIHWYQQKPGQPPTLLIQLASNVQTGVPARFSGSGSRTDFTLTIDPVEEDDVAVYYCLQSRTIPRTFGGGTKLEIKGGGGSGGGGSGGGGSQIQLVQSGPELKKPGETVKISCKASGYTFTDYSINWVKRAPGKGLKWMGWINTETREPAYAYDFRGRFAFSLETSASTAYLQINNLKYEDTATYFCALDYSYAMDYWGQGTSVTVSSGGGGSGGGGSGGGGSDKTHTCPPCPAPEAAGGPSVFLFPPKPKDTLMISRTPEVTCVVVDVSHEDPEVKFNWYVDGVEVHNAKTKPREEQYNSTYRVVSVLTVLHQDWLNGKEYKCKVSNKALPAPIEKTISKAKGQPREPQVYTLPPCRDELTKNQVSLWCLVKGFYPSDIAVEWESNGQPENNYKTTPPVLDSDGSFFLYSKLTVDKSRWQQGNVFSCSVMHEALHNHYTQKSLSLSPG

### Monovalent (iTCR scFV-IgG) AA sequences

MPLLLLLPLLWAGALADIKMTQSPSSMYASLGERVTITCKASQDINSYLSWFQQKAGKSPKTLIYRANRLVDGVPSRFSGSGSGQDYSLTISSLEYEDMGIYYCLQYDEFPFTFGGGTRLEIKGGGGSGGGGSGGGGSQVQLQQSGPEVVRPGVSVKISCKGSGYRFTDSAMHWVKQSHAKSLEWIGVISSYNGNTNYNQKFKGKATMTVDKSSSTAYMELARMTSEDSAIYYCARSRDAMDYWGQGTSVTVSSGGGGSGGGGSGGGGSDKTHTCPPCPAPEAAGGPSVFLFPPKPKDTLMISRTPEVTCVVVDVSHEDPEVKFNWYVDGVEVHNAKTKPREEQYNSTYRVVSVLTVLHQDWLNGKEYKCKVSNKALPAPIEKTISKAKGQPREPQVYTLPPCRDELTKNQVSLTCLVKGFYPSDIAVEWESNGQPENNYKTTPPVLDSDGSFFLTSKLTVDKSRWQQGNVFSCSVMHEALHNHYTQKSLSLSPG

### Anti-BCMA FAB AA sequences

#### Heavy chain

QIQLVQSGPELKKPGETVKISCKASGYTFTDYSINWVKRAPGKGLKWMGWINTETREPAYAYDFRGRFAFSLETSASTAYLQINNLKYEDTATYFCALDYSYAMDYWGQGTSVTVSS

#### Light chain

DIVLTQSPPSLAMSLGKRATISCRASESVTILGSHLIHWYQQKPGQPPTLLIQLASNVQTGVPARFSGSGSRTDFTLTIDPVEEDDVAVYYCLQSRTIPRTFGGGTKLEIK

### Fc IG AA sequences

ASTKGPSVFPLAPSSKSTSGGTAALGCLVKDYFPEPVTVSWNSGALTSGVHTFPAVLQSSGLYSLSSVVTVPSSSLGTQTYICNVNHKPSNTKVDKKVEPKSCDKTHTCPPCPAPEAAGGPSVFLFPPKPKDTLMISRTPEVTCVVVDVSHEDPEVKFNWYVDGVEVHNAKTKPREEQYNSTYRVVSVLTVLHQDWLNGKEYKCKVSNKALPAPIEKTISKAKGQPREPQVYTLPPSRDELTKNQVSLTCLVKGFYPSDIAVEWESNGQPENNYKTTPPVLDSDGSFFLYSKLTVDKSRWQQGNVFSCSVMHEALHNHYTQKSLSLS

### Immunofluorescence and confocal microscopy

For immune synapse imaging, 10^6^/ml of untransduced iNKT or BCMA CAR-iNKT cells were mixed with MM1.S target cells at a 1:1 ratio in serum-free RPMI and incubated for 30–45 min at 37 °C on a glass microscope slide (#PH299 BK, Hendley Essex). Cells were fixed in ice-cold methanol for 5 min, blocked in 2% BSA in PBS for 40 min and stained overnight at 4 °C with rabbit anti-actin (cat no. A2066, MilliporeSigma) and mouse anti-γTubulin (cat no. T6557, MilliporeSigma) primary antibodies. The next day, samples were washed and incubated with a donkey anti-rabbit (cat no. A-31572, ThermoFisher) or a goat anti-mouse (cat no. A-11001, ThermoFisher) fluorophore-conjugated secondary antibodies. For PLXND1 expression analysis, iNKT cells only were incubated on the slides for 15 min at 37 °C, fixed for 15 min in 4% paraformaldehyde (cat no. 15710-S, Electron Microscopy Systems) and permeabilized in 0.1% Triton X-100 (Sigma) for 5 min. Samples were then blocked and stained as described above using a primary anti-Plexin D1 antibody (cat no. ab28762; Abcam) followed by a goat anti-rabbit fluorophore-conjugated secondary antibody (cat no. A-21428; ThermoFisher) and Alexa Fluor® 488 phalloidin (cat no. 8878; Cell Signaling Technology) for 45 min at RT. All samples were mounted in antifade medium with DAPI (cat no. H-1500-10; Vector Laboratories) and were acquired on an LSM-780 confocal laser scanning microscope. Z-stack series of 0.62 μm intervals were taken with a ×63 oil DIC Plan-Apochromat objective (numerical aperture 1.4).

### Data analysis and software

All the flow cytometry analysis was performed using FlowJo software. The graphical data and statistics were performed using Prism 10 software and BioRender tools under Imperial academic licence. Unpaired t-test or non-parametric Mann–Whitney *U* test or ordinary one-way ANOVA were used to compare independent groups, and significance were calculated based on the *p* values. For the line graphs, the significance color code matches the sample color in the graph. All the RNA sequencing traces were aligned using STAR, and differential analysis was performed using DESeq2 R Bioconductor packages by paired analysis. ImageJ was used for automatic cell counting of bound cells on monolayers for cell avidity experiments. In vivo Imaging System (IVIS) and Aura software were used for in vivo bioluminescence photons analysis. PLXND1 expression in immune cells was derived from the Human Protein Atlas. The original datasets from which the relevant data were curated were from Monaco et al.^24^ and Schmiedel et al.^25^.

### Reporting summary

Further information on research design is available in the Nature Portfolio Reporting Summary linked to this article.

## Supplementary information

Supplementary Information

Description of Additional Supplementary Files

Supplementary Dataset 1

Reporting Summary

Transparent Peer Review File

## Data Availability

The RNA sequencing data has been submitted to the GEO database (accession #GSE312032) https://www.ncbi.nlm.nih.gov/geo/query/acc.cgi?acc=GSE312032, and all the associated data has been submitted in the Supplementary files and Source data spreadsheets shared in the figshare repository https://doi.org/10.6084/m9.figshare.32390247. Public data sets for Supplementary Fig. 2C, D^46^^,^^47^ were derived from the Human Protein Atlas database https://www.proteinatlas.org/ENSG00000004399-PLXND1/single+cell. SEMA4A expression for MM1S and H929 cells (Fig. 2G and Supplementary Fig. 2F) was derived from CCLE data sets https://sites.broadinstitute.org/ccle/datasets, and MMRF data sets for primary cells (Supplementary Fig. 2G, H) https://themmrf.org/for-researchers/data-access-via-virtual-lab/.
