# Supplementary material for: Enhancing iNKT cell immunotherapy through the integration of optimized CAR endodomains and iNKT engagers
Source: Nat Commun. 2026 Aug 14;17:9755. doi: 10.1038/s41467-026-76718-7 (PMC13572498; doi:10.1038/s41467-026-76718-7)
Supplement: Supplementary file 1 [file 41467_2026_76718_MOESM1_ESM.pdf]

Supplementary information for the article entitled

Enhancing the iNKT cell immunotherapy platform by combining optimised CAR endodomains with novel iNKT engagers

Kanagaraju Ponnusamy[1,2]*✉, Klesti Karaxhuku[1,2]*, Yuchao Jiang[1], Lyra Randzavola[2,3], Hongwei Ren[2], Ilia Leontari[1,2], Bryan Lye[2], Mehmood Zaidi[1,2], Edward J Bartlett[4], Edward W Tate[4], Vasileios Pardalis[2], Dimitrios Leonardos[1,5], Reza Nadafi[6], Irene Sarkar[6], Rogier M Reijmers[6], Marco Bua[7], Maria Atta[7], Alexia Katsarou[1,7], Irene AG Roberts[8,9], Aristeidis Chaidos[1,2,7], Anastasios Karadimitris[1,2,7]✉

[1]Hugh & Josseline Langmuir Centre for Myeloma Research Centre for Haematology, Department of Immunology and Inflammation, Imperial College London, London, United Kingdom.

[2]Centre for Haematology, Department of Immunology and Inflammation, Imperial College London, London, United Kingdom.

[3]Centre for Inflammatory Disease, Department of Immunology and Inflammation, Imperial College London, London, United Kingdom.

[4]Department of Chemistry, Natural Sciences, Imperial College London, London, United Kingdom.

[5]University of Ioannina Medical School, Ioannina, Greece.

[6]Lumicks, Amsterdam, Netherlands.

[7]Department of Haematology, Hammersmith Hospital, Imperial College Healthcare NHS Trust, London, United Kingdom.

[8]Department of Paediatrics, University of Oxford, Oxford, United Kingdom & [9]Molecular Haematology Unit, MRC Weatherall Institute of Molecular Medicine, University of Oxford, Oxford, United Kingdom.

Correspondence

Anastasios Karadimitris a.karadimitris@imperial.ac.uk

Kanagaraju Ponnusamy k.ponnusamy@imperial.ac.uk

Suppl Fig. 1

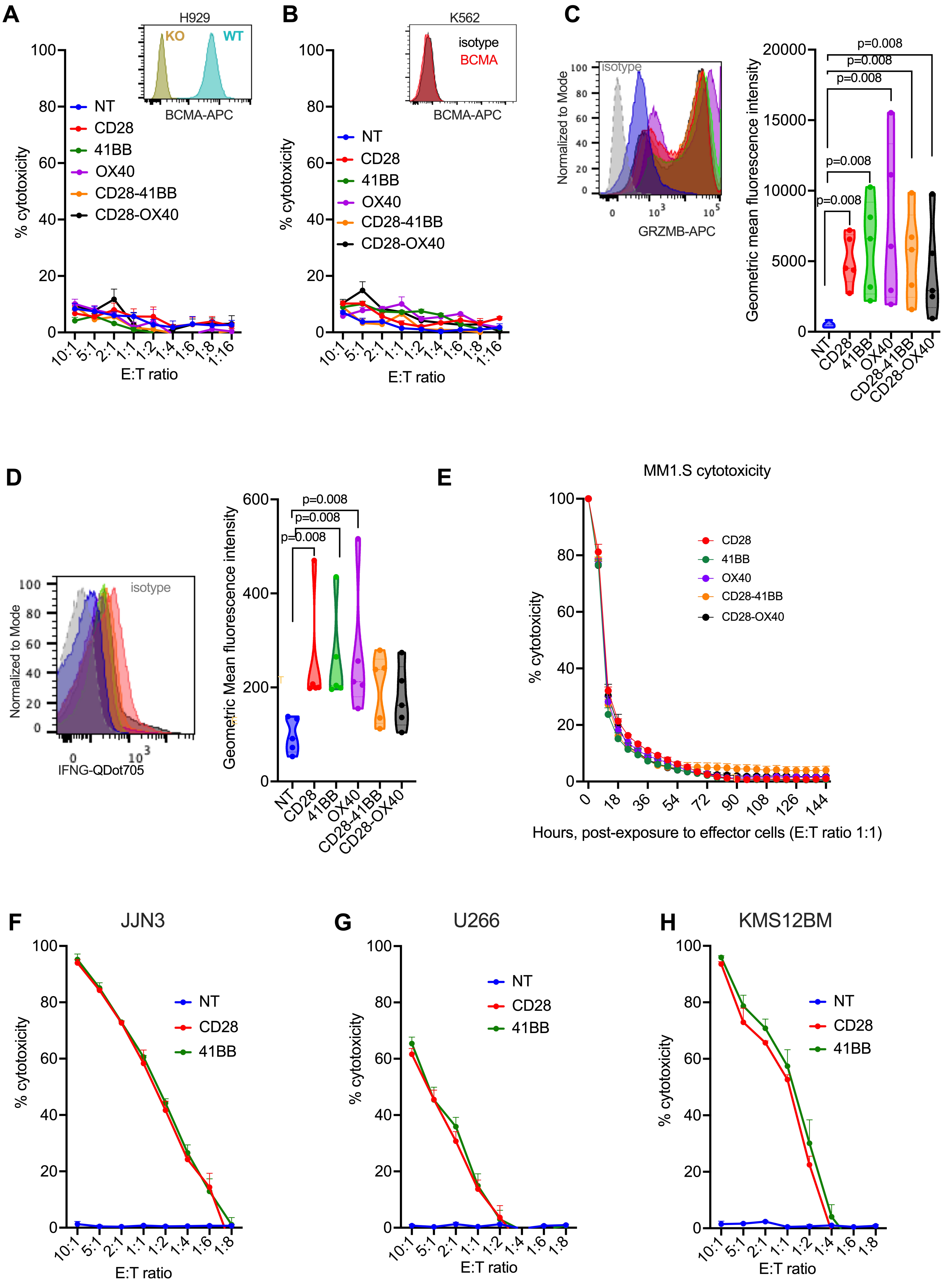

A
H929
KO
WT
BCMA-APC
% cytotoxicity
NT
CD28
41BB
OX40
CD28-41BB
CD28-OX40
E:T ratio
B
K562
isotype
BCMA
C
isotype
Normalized to Mode
GRZMB-APC
Geometric mean fluorescence intensity
p=0.008
D
IFNG-QDot705
Geometric Mean fluorescence intensity
E
MM1.S cytotoxicity
Hours, post-exposure to effector cells (E:T ratio 1:1)
F
JJN3
G
U266
H
KMS12BM

Suppl Fig. 1

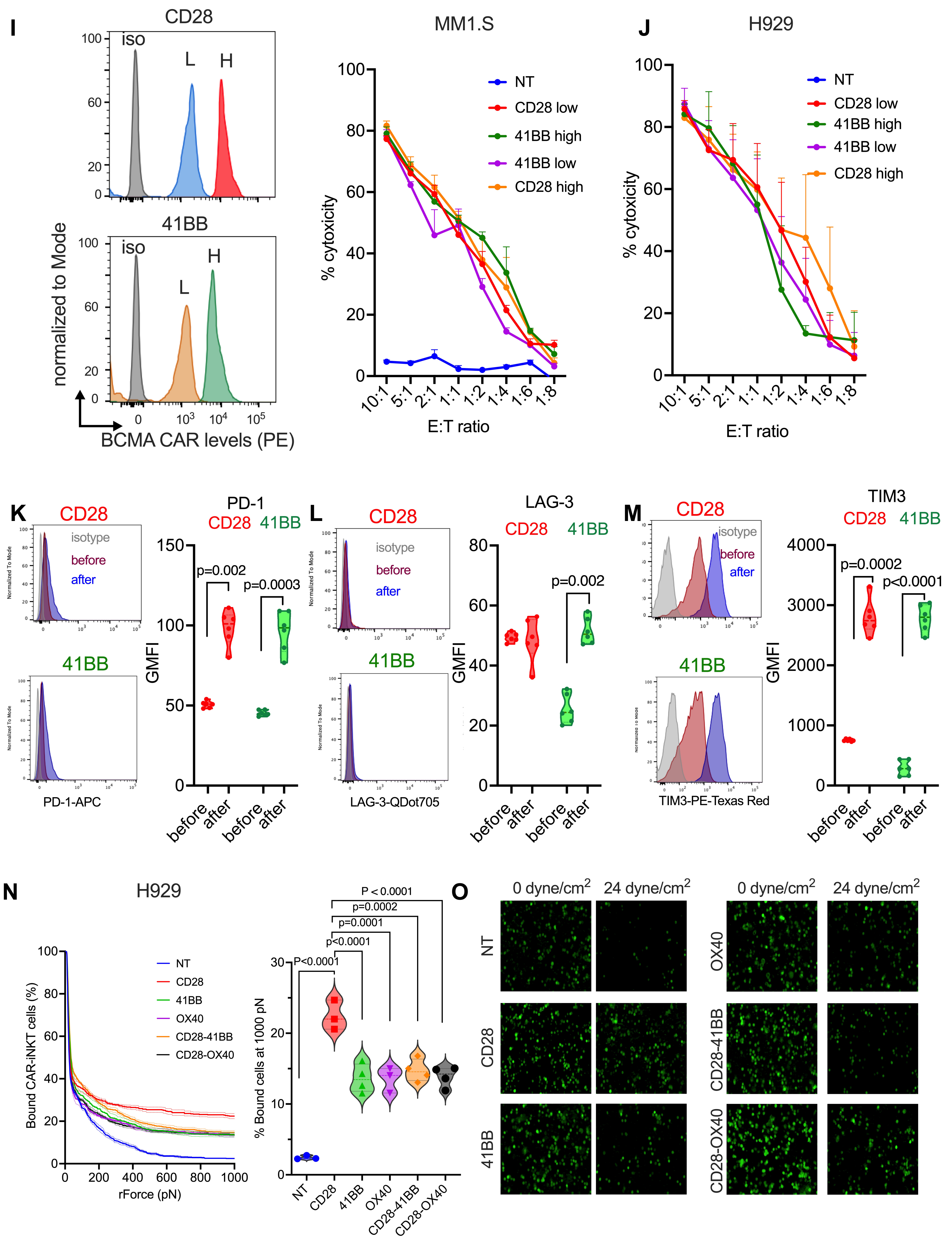
I
CD28
iso
L
H
41BB
iso
L
H
normalized to Mode
BCMA CAR levels (PE)
MM1.S
% cytoxicity
NT
CD28 low
41BB high
41BB low
CD28 high
E:T ratio
10:1
5:1
2:1
1:1
1:2
1:4
1:6
1:8
J
H929
K
CD28
isotype
before
after
41BB
PD-1-APC
PD-1
p=0.002
p=0.0003
GMFI
L
LAG-3-QDot705
LAG-3
M
TIM3-PE-Texas Red
TIM3
p=0.0002
p<0.0001
N
H929
Bound CAR-iNKT cells (%)
rForce (pN)
NT
CD28
41BB
OX40
CD28-41BB
CD28-OX40
% Bound cells at 1000 pN
P < 0.0001
p=0.0002
p=0.0001
p<0.0001
P<0.0001
O
0 dyne/cm²
24 dyne/cm²

Supplementary Figure 1 (related to Figure 1)

A. BCMA expression in BCMA WT and BCMA KO H929 cells shown in histogram and their cytotoxicity by CAR iNKT cells at different E:T ratio. (n=4 donors).

B. BCMA expression in K562 erythromyeloid cells shown in histogram and CAR iNKT cell-mediated cytotoxicity against the same cells. (n=3 donors).

C. Left: Intracellular expression of granzyme B levels in CAR-iNKT measured by flow cytometry after exposing cells to the MM1.S myeloma cells for 16hours. Right: cumulative data from four different donors.

D. Left: Intracellular expression of interferon gamma levels in CAR-iNKT measured by flow cytometry after exposing the cells to the MM1.S myeloma cells for 16hours. Right: cumulative data from four different donors.

E. Cytotoxicity of MM1.S cells, that express tdTomato fluorescence marker, by BCMA CAR iNKT cells with different co-stimulatory molecules assessed by real-time Incucyte® imaging. The cytotoxicity levels determined by tdTomato florescence signals and normalized to hour 0 (n=duplicates of 3 donors).

F-H. Comparison of cytotoxic activity of CD28z and 41BBz CAR iNKT cells against JJN3 (F), U266 (G) and KMS12BM (H) myeloma cells is shown. (n=3 donors)

I-J. After CD28z and 41BBz BCMA CAR iNKT cells were FACS-sorted for low (L) and high (H) levels of CAR expression (I); their cytotoxic activity against MM1.S cells (I) and H929 cells (J) was tested. iso-isotype, L-low BCMA CAR iNKT, H-high BCMA CAR iNKT, NT-non-transduced iNKT cells. (n=3 donors).

K-M. PD-1 expression levels (K), LAG-3 (L) and TIM-3 (M) expression levels before exposing to target cells MM1.S and after exposing for 4 challenges between CD28z and 41BBz CAR iNKT cells. (n=duplicates of 3 donors). The p value calculated by the mean of each duplicates.

N. Left: CD28 CAR iNKT cell binding activity on H929 myeloma cells under increasing levels of acoustic force as compared to all other CAR- and NT iNKT; right: CAR-iNKT binding at rForce 1000 pN (data representative of n=2 donors).

O. Representative images of cell binding before shear stress (0 dyne/cm^2) and after 24 dyne/cm^2 which are related to Figure 1K. Two-tailed statistical analysis Mann-Whitney U test (C & D), unpaired t-test (K-M) and ordinary one-way Anova (N) performed. All error bars show Mean±SEM, and box and whiskers show minimum to maximum values with all data points. p <0.0001 is referred as $p < 0.0001$. non significance measured by $p > 0.05$.

Suppl Fig. 2

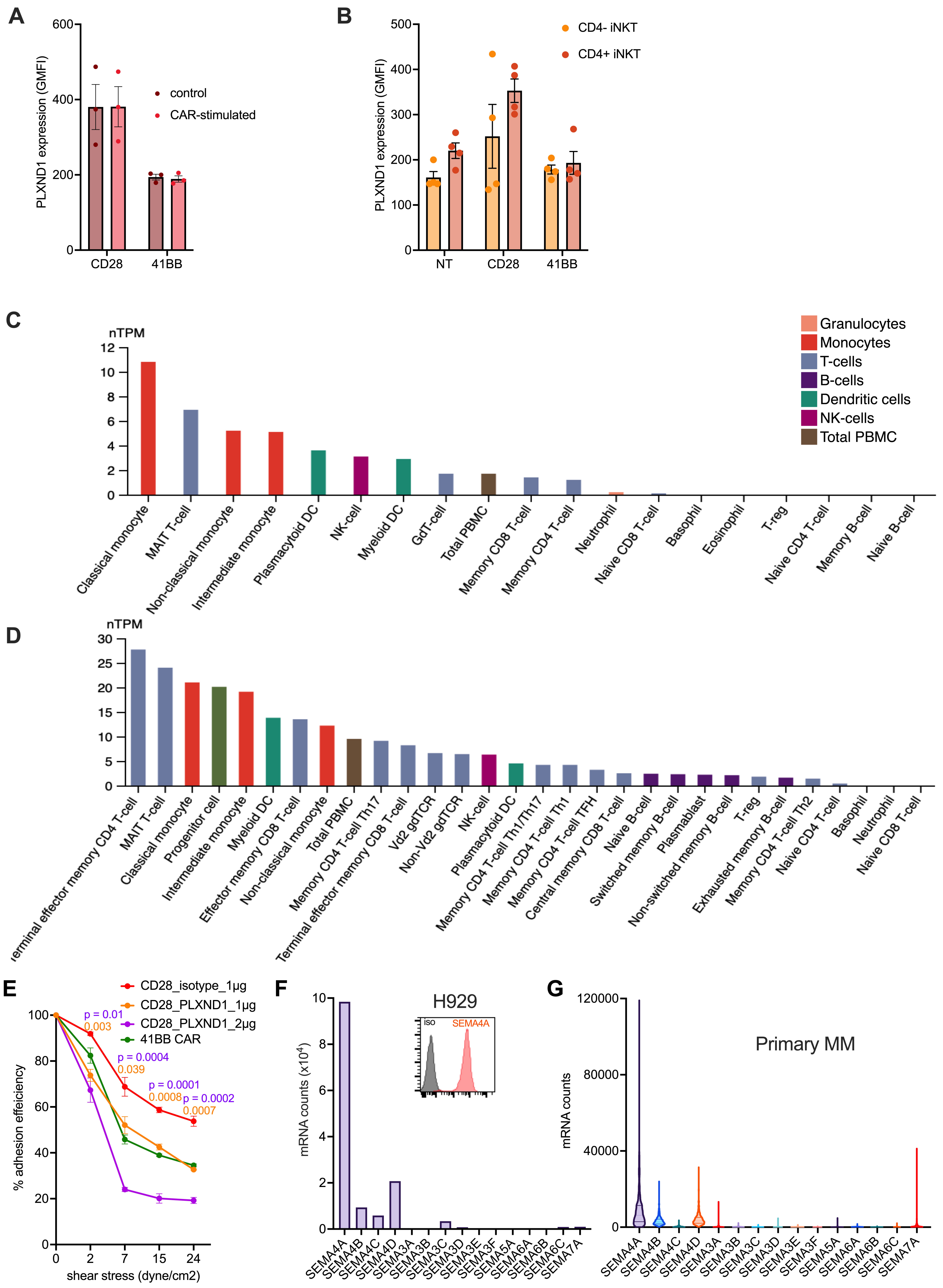
A
PLXND1 expression (GMFI)
control
CAR-stimulated
CD28
41BB
B
PLXND1 expression (GMFI)
CD4- iNKT
CD4+ iNKT
NT
CD28
41BB
C
nTPM
Granulocytes
Monocytes
T-cells
B-cells
Dendritic cells
NK-cells
Total PBMC
Classical monocyte
MAIT T-cell
Non-classical monocyte
Intermediate monocyte
Plasmacytoid DC
NK-cell
Myeloid DC
GdT-cell
Total PBMC
Memory CD8 T-cell
Memory CD4 T-cell
Neutrophil
Naive CD8 T-cell
Basophil
Eosinophil
T-reg
Naive CD4 T-cell
Memory B-cell
Naive B-cell
D
nTPM
Terminal effector memory CD4 T-cell
MAIT T-cell
Classical monocyte
Progenitor cell
Intermediate monocyte
Myeloid DC
Effector memory CD8 T-cell
Non-classical monocyte
Total PBMC
Memory CD4 T-cell Th17
Terminal effector memory CD8 T-cell
Vd2 gdTCR
Non-Vd2 gdTCR
NK-cell
Plasmacytoid DC
Memory CD4 T-cell Th1/Th17
Memory CD4 T-cell Th1
Memory CD4 T-cell TFH
Central memory CD8 T-cell
Naive B-cell
Switched memory B-cell
Plasmablast
Non-switched memory B-cell
T-reg
Exhausted memory B-cell
Memory CD4 T-cell Th2
Naive CD4 T-cell
Basophil
Neutrophil
Naive CD8 T-cell
E
CD28_isotype_1μg
CD28_PLXND1_1μg
CD28_PLXND1_2μg
41BB CAR
p = 0.01
0.003
p = 0.0004
0.039
p = 0.0001
0.0008
p = 0.0002
0.0007
% adhesion effeiciency
shear stress (dyne/cm2)
F
H929
iso
SEMA4A
mRNA counts (x10⁴)
SEMA4A
SEMA4B
SEMA4C
SEMA4D
SEMA3A
SEMA3B
SEMA3C
SEMA3D
SEMA3E
SEMA3F
SEMA5A
SEMA6A
SEMA6B
SEMA6C
SEMA7A
G
Primary MM
mRNA counts
SEMA4A
SEMA4B
SEMA4C
SEMA4D
SEMA3A
SEMA3B
SEMA3C
SEMA3D
SEMA3E
SEMA3F
SEMA5A
SEMA6A
SEMA6B
SEMA6C
SEMA7A

Suppl Fig. 2

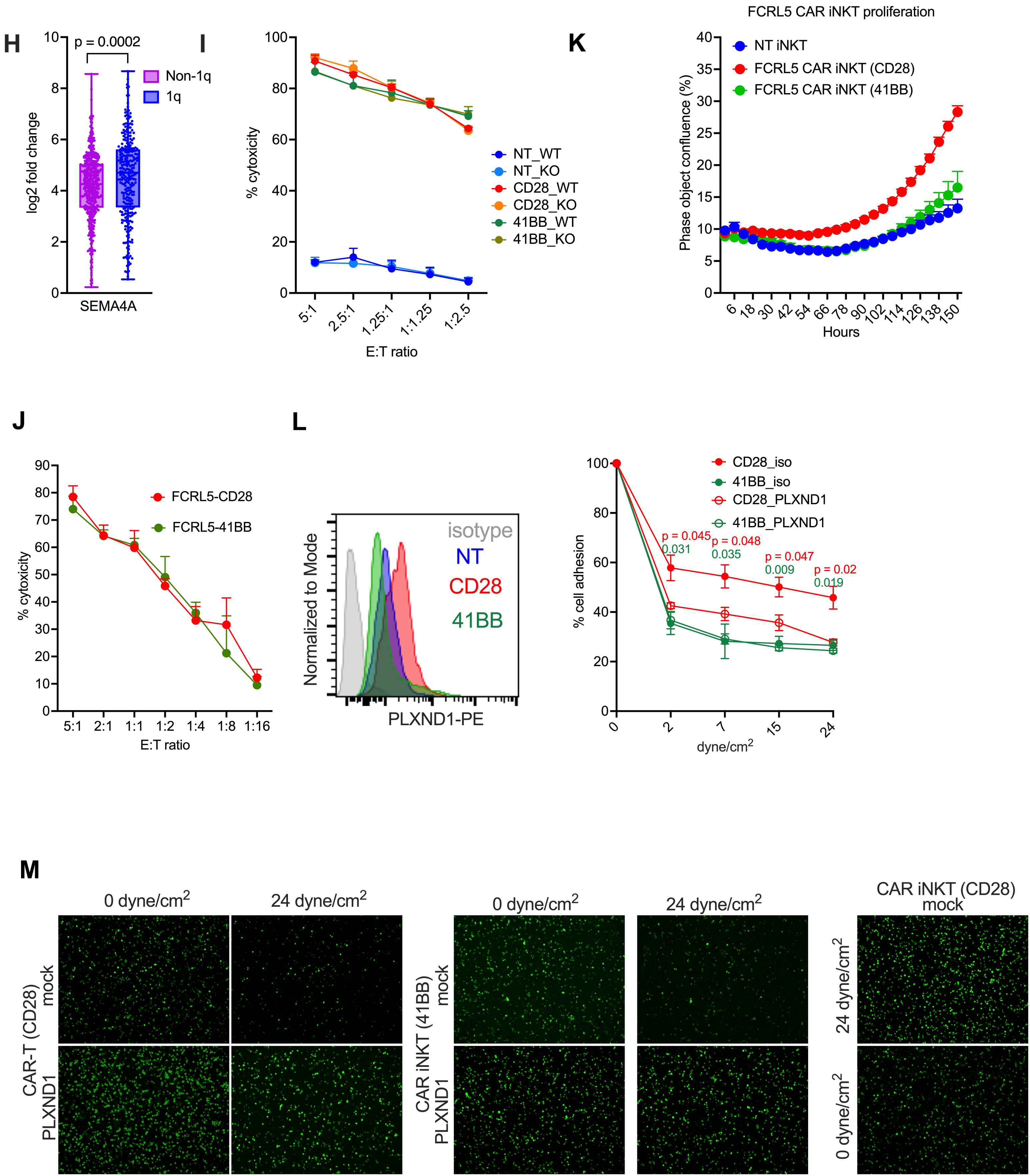

H
p = 0.0002
Non-1q
1q
log2 fold change
SEMA4A
I
% cytoxicity
NT_WT
NT_KO
CD28_WT
CD28_KO
41BB_WT
41BB_KO
E:T ratio
5:1
2.5:1
1.25:1
1:1.25
1:2.5
K
FCRL5 CAR iNKT proliferation
NT iNKT
FCRL5 CAR iNKT (CD28)
FCRL5 CAR iNKT (41BB)
Phase object confluence (%)
Hours
J
% cytoxicity
FCRL5-CD28
FCRL5-41BB
E:T ratio
5:1
2:1
1:1
1:2
1:4
1:8
1:16
L
Normalized to Mode
isotype
NT
CD28
41BB
PLXND1-PE
CD28_iso
41BB_iso
CD28_PLXND1
41BB_PLXND1
% cell adhesion
p = 0.045
0.031
p = 0.048
0.035
p = 0.047
0.009
p = 0.02
0.019
dyne/cm2
M
0 dyne/cm2
24 dyne/cm2
CAR-T (CD28)
mock
PLXND1
CAR iNKT (41BB)
CAR iNKT (CD28)

Supplementary Figure 2 (related Figure 2)

A. PLXND1 expression in CD28z CAR iNKT cells as assessed by flow cytometry after stimulation with MM1.S myeloma cells for 24hr (n=3).

B. PLXND1 expression in CD4+ and CD4- fractions of iNKT cells as assessed by flow cytometry shown as GMFI (n=4).

C-D. Expression of PLXND1 in subpopulations of immune cells. Data derived from Human Protein Atlas database[46,47].

E. Dose-dependent reduction of avidity of BCMA CAR iNKT with CD28z co-stimulatory molecules by PLXND1 blocking antibody. BCMA CAR with 41BBz shown as control for 2 donors.

F. SEMA4A mRNA count values and its family members in H929 cells (CCLE Dataset). The histogram shows SEMA4A expression on the surface of H929 cells.

G. Expression of SEMA4A mRNA counts and its family members in 770 primary myeloma cells from the MMRF CoMMpass study.

H. SEMA4A expression is higher in Chr 1q+ primary MM cells as compared to non-1q MM cells (MMRF CoMMpass Dataset).

I. No difference in cytotoxicity by BCMA CAR iNKT cells observed against SEMA4+ MM1.S (WT) and SEMA4- MM1.S (KO) cells (n=3 donors).

J. No difference in cytotoxic activity between FCRL5-CD28z and FCRL5-41BBz CAR iNKT cells against MM1.S-FCRL5+ target cells.

K. FCRL5-CD28z CAR iNKT cells shows higher proliferative potential than its 41BBz counterpart and NT iNKT cells in short-term proliferation assay in the presence of IL-15 as assessed by real-time Incucyte® imaging (n=3 donors). Unpaired t test shows p<0.001 for CD28 compared to NT and 41BB from 60hr.

L. Left: Flow cytometry analysis of Plexin D1 expression in FCRL5 CAR iNKT cells with CD28z or 41BBz costimulatory molecules as compared to NT iNKT cells. Right: cell avidity of FCRL5-CD28z and FCRL5-41BBz CAR iNKT cells on MM1.S-FCRL5+ cells under shear stress with isotype control or PLXND1-blocking antibody at 1µg/ml concentration (n=3).

M. Representative images of indicated effectors bound to MM1.S myeloma cells at 0 and 24 dyne/cm^2 which are related Figure 2I. Two-tailed statistical analysis Mann-Whitney U test (B, H) and unpaired t test (E & L) performed. All error bars show Mean±SEM, and box and whiskers show minimum to maximum values with all data points. p <0.0001 is referred as p < 0.0001. non significance measured by p > 0.05.

Suppl Fig. 3

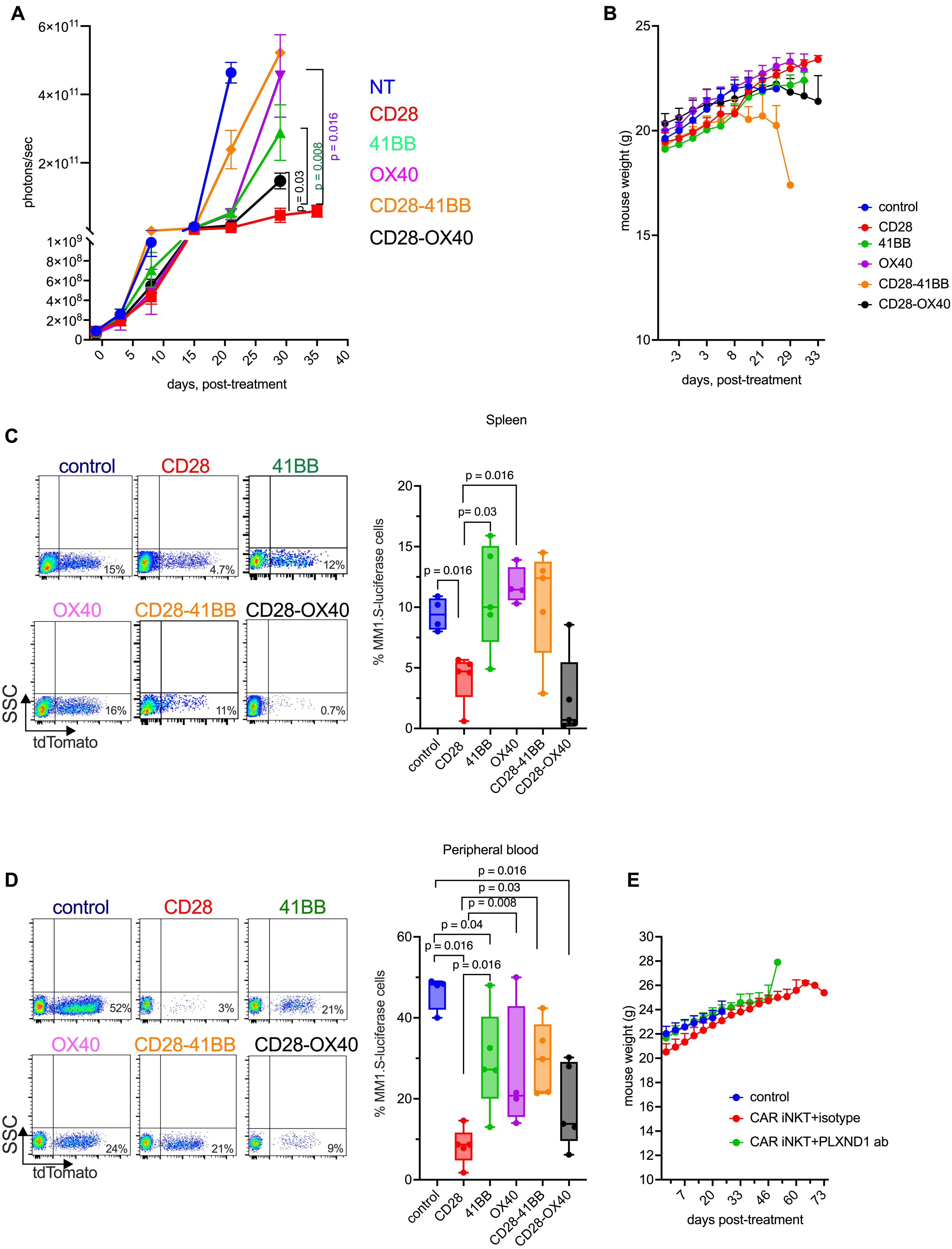

A
photons/sec
days, post-treatment
NT
CD28
41BB
OX40
CD28-41BB
CD28-OX40
p = 0.03
p = 0.008
p = 0.016
B
mouse weight (g)
days, post-treatment
control
CD28
41BB
OX40
CD28-41BB
CD28-OX40
C
Spleen
control
CD28
41BB
OX40
CD28-41BB
CD28-OX40
15%
4.7%
12%
16%
11%
0.7%
SSC
tdTomato
% MM1.S-luciferase cells
p = 0.016
p= 0.03
p = 0.016
D
Peripheral blood
control
CD28
41BB
OX40
CD28-41BB
CD28-OX40
52%
3%
21%
24%
21%
9%
SSC
tdTomato
% MM1.S-luciferase cells
p = 0.016
p = 0.03
p = 0.008
p = 0.04
p = 0.016
p = 0.016
E
mouse weight (g)
days post-treatment
control
CAR iNKT+isotype
CAR iNKT+PLXND1 ab

Supplementary Figure 3 (related Figure 3)

A. Cumulative photon strength of total body in animals treated with BCMA CAR iNKT with different co-stimulatory domains as also shown in Figure 3C (n=4-5 mice per group).

D. Body weights of the mice treated with CAR iNKT cells shown in Figure 3A-H (n=4-5 mice per group).

C-D. Frequency of MM1.S-luci tumor cells in the spleen (C), peripheral blood at end-point (D) (n=4-5 mice per group) as measured by tdTomato fluorescence marker using flow cytometry.

E. Body weights of the mice treated with CAR iNKT cells shown in Figure 3I-K (n=4-6 mice per group).

Two-tailed statistical analysis Mann-Whitney U test (A, C & D) performed. All error bars show Mean±SEM, and box and whiskers show minimum to maximum values with all data points. $p <0.0001$ is referred as $p < 0.0001$. non significance measured by $p > 0.05$.

Suppl Fig. 4

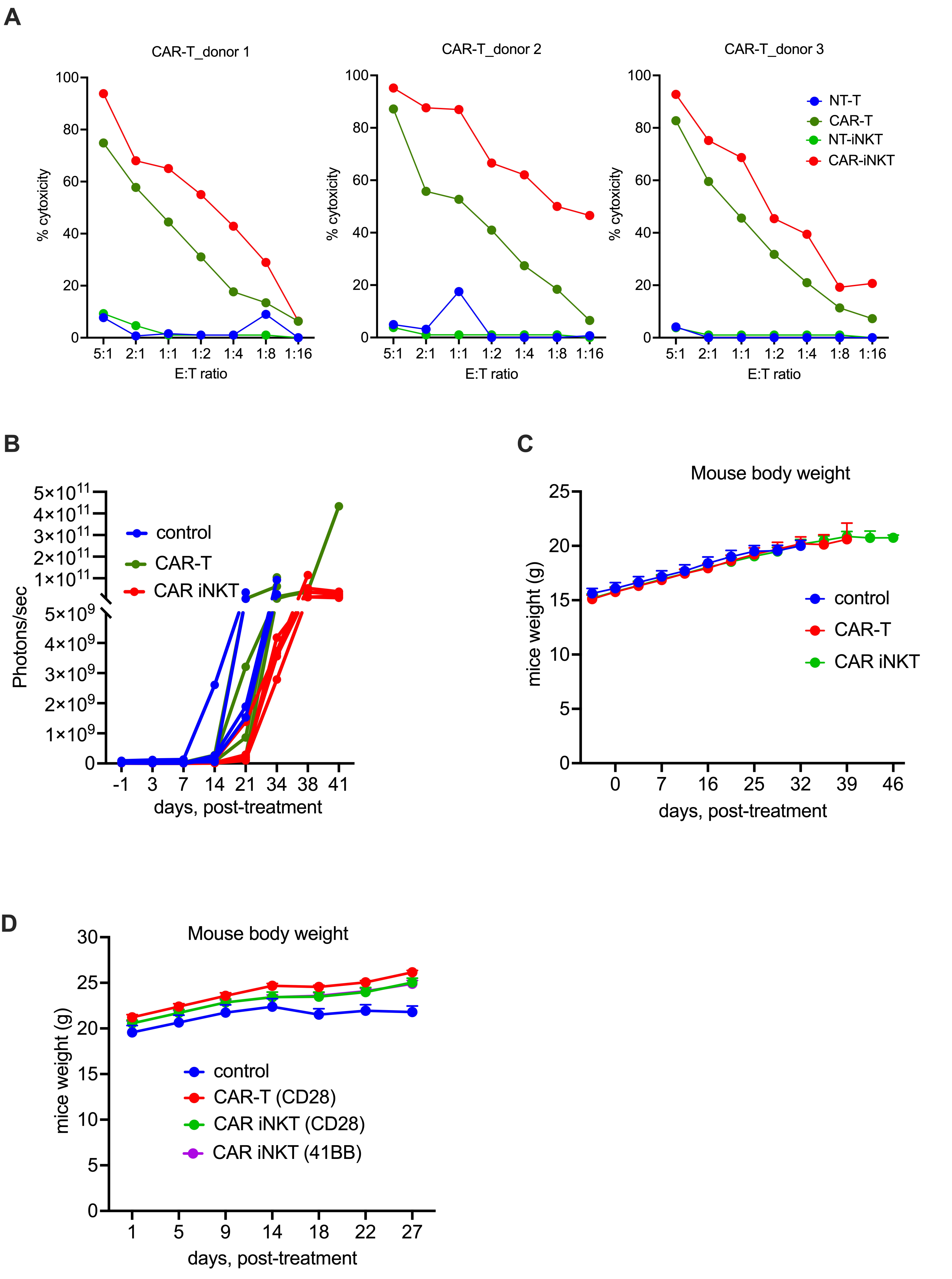
A
CAR-T_donor 1
CAR-T_donor 2
CAR-T_donor 3
% cytoxicity
100
80
60
40
20
0
5:1
2:1
1:1
1:2
1:4
1:8
1:16
E:T ratio
NT-T
CAR-T
NT-iNKT
CAR-iNKT
B
Photons/sec
5×10^11
4×10^11
3×10^11
2×10^11
1×10^11
5×10^9
4×10^9
3×10^9
2×10^9
1×10^9
0
-1
3
7
14
21
34
38
41
days, post-treatment
control
CAR-T
CAR iNKT
C
Mouse body weight
mice weight (g)
25
20
15
10
5
0
0
7
16
25
32
39
46
days, post-treatment
control
CAR-T
CAR iNKT
D
Mouse body weight
mice weight (g)
30
25
20
15
10
5
0
1
5
9
14
18
22
27
days, post-treatment
control
CAR-T (CD28)
CAR iNKT (CD28)
CAR iNKT (41BB)

Supplementary Figure 4 (related for Figure 4)

A. Cytotoxic potential of same donor BCMA-CD28z CAR iNKT and BCMA-CD28z CAR-T from 3 different donors against MM1.S cells.

B. Cumulative total body BLI signals shown for figure 4D.

C&D. Longitudinal measurement of weight of mice described in Figure 4A-G (C) and 4H-J (D).

All figure's error bars show Mean± SEM. $p < 0.0001$ is referred as $p < 0.0001$. non significance measured by $p > 0.05$.

Suppl Fig. 5

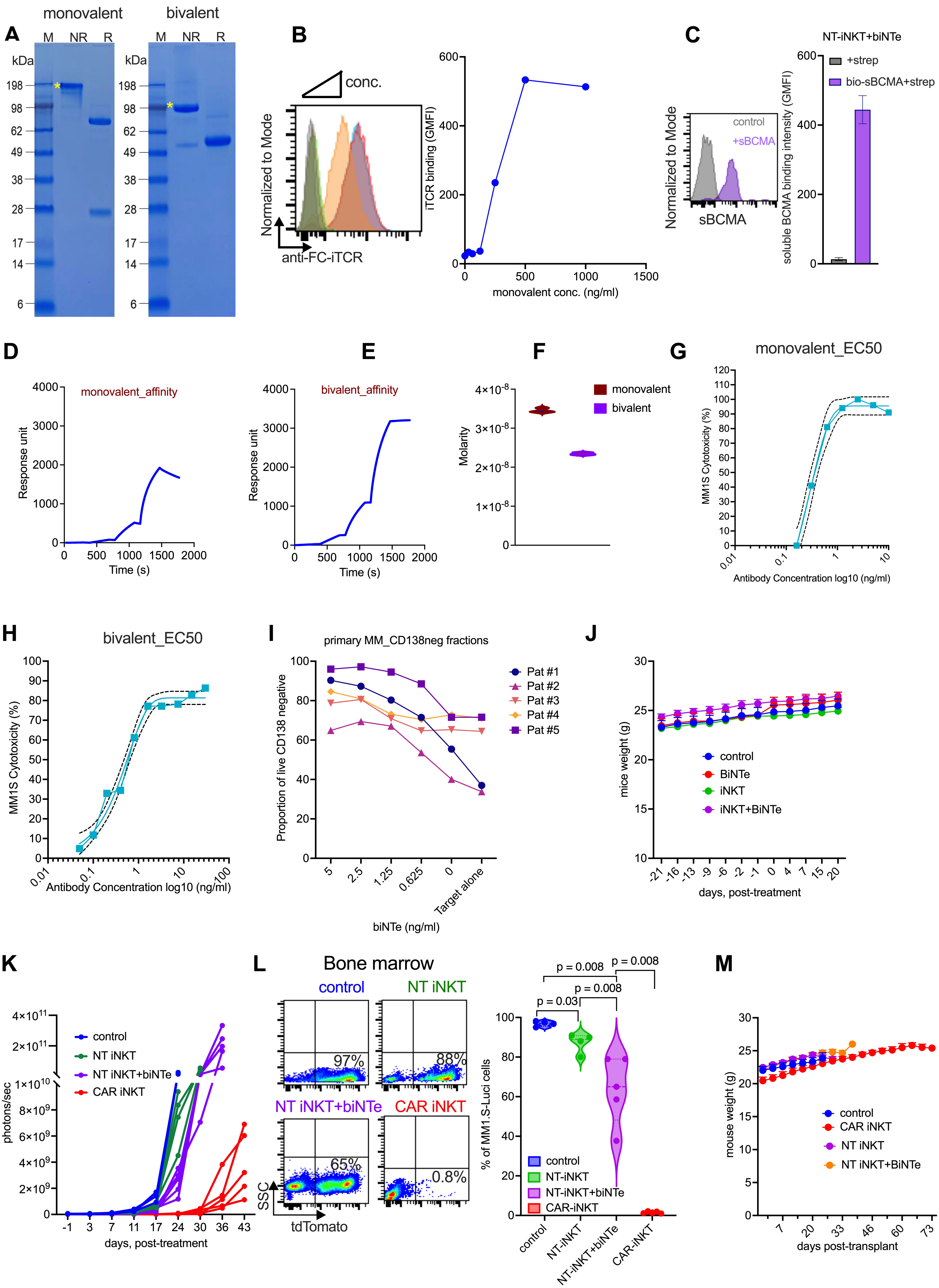

A
monovalent
bivalent
M NR R
kDa
198
98
62
49
38
28
17
14
6
B
conc.
Normalized to Mode
anti-FC-iTCR
iTCR binding (GMFI)
monovalent conc. (ng/ml)
C
NT-iNKT+biNTe
control
+sBCMA
sBCMA
+strep
bio-sBCMA+strep
soluble BCMA binding intensity (GMFI)
D
monovalent_affinity
Response unit
Time (s)
E
bivalent_affinity
F
monovalent
bivalent
Molarity
G
monovalent_EC50
MM1S Cytotoxicity (%)
Antibody Concentration log10 (ng/ml)
H
bivalent_EC50
I
primary MM_CD138neg fractions
Proportion of live CD138 negative
Pat #1
Pat #2
Pat #3
Pat #4
Pat #5
Target alone
biNTe (ng/ml)
J
mice weight (g)
control
BiNTe
iNKT
iNKT+BiNTe
days, post-treatment
K
photons/sec
control
NT iNKT
NT iNKT+biNTe
CAR iNKT
L
Bone marrow
control
NT iNKT
NT iNKT+biNTe
CAR iNKT
97%
88%
65%
0.8%
SSC
tdTomato
p = 0.008
p = 0.008
p = 0.03
p = 0.008
% of MM1.S-Luci cells
NT-iNKT
NT-iNKT+biNTe
CAR-iNKT
M
mouse weight (g)
days post-transplant

Supplementary Figure 5 (related to Figure 5)

A. SDS-PAGE gel analysis of purity of monovalent (left) and bivalent biNTe (right) under reducing (R) and non-reducing (NR). M indicates marker. * Indicates the band size ~198.9 kDa for monovalent and ~78.9 kDa for bivalent biNTe under non-reducing conditions.

B. Increasing levels of binding efficiency at increasing level of antibody concentration (0-1µg) for monovalent antibody to iNKT cells. Concentration range (0-1µg/ml).

C. Flow-cytometric analysis of pure iNKT cells after staining with bivalent biNTe and fluorescently labelled streptavidin (control: grey) or with bivalent biNTe, biotinylated soluble BCMA and fluorescently labelled streptavidin (purple). Representative histogram (left) and its cumulative data (right) shown as GMFI. (n=3 donors).

D-F. Affinity strength of monovalent (D), bivalent (E) antibodies on soluble BCMA against indicated time and their kd values (F) as determined in SPR assay, (n=3).

G&H. EC50 values shown for monovalent (G) and bivalent engagers (H) against MM1.S by co-culture of NT iNKT at 1:1 E:T ratio plus indicated engagers. EC50 values were calculated by using non-linear fit, log(agonist) vs. response function in prism 10 software.

I. CD138 negative fractions of primary myeloma cells after incubating with NT iNKT cells and biNTe as part of the CD138+ fractions analysis shown in Figure 5G.

J. Body weight measurement for mice shown in Figure 5H.

K. Cumulative BLI data for mice treated with NT iNKT and/or biNTe, and CAR iNKT and control group as shown in Figure 5J-L. (n=4-5 mice per group).

L. Flow cytometric analysis for frequency of MM1.S cells in the bone marrow from each group at end point. Left: representative flow cytometry plots; right: cumulative data. (n=4-5 mice per group).

M. Body weight measurement for mice shown in Figure 5J-L.

Two-tailed statistical analysis Mann-Whitney U test (L) performed. All error bars show Mean±SEM. $p < 0.0001$ is referred as $p < 0.0001$. non significance measured by $p > 0.05$.

Suppl Fig. 6

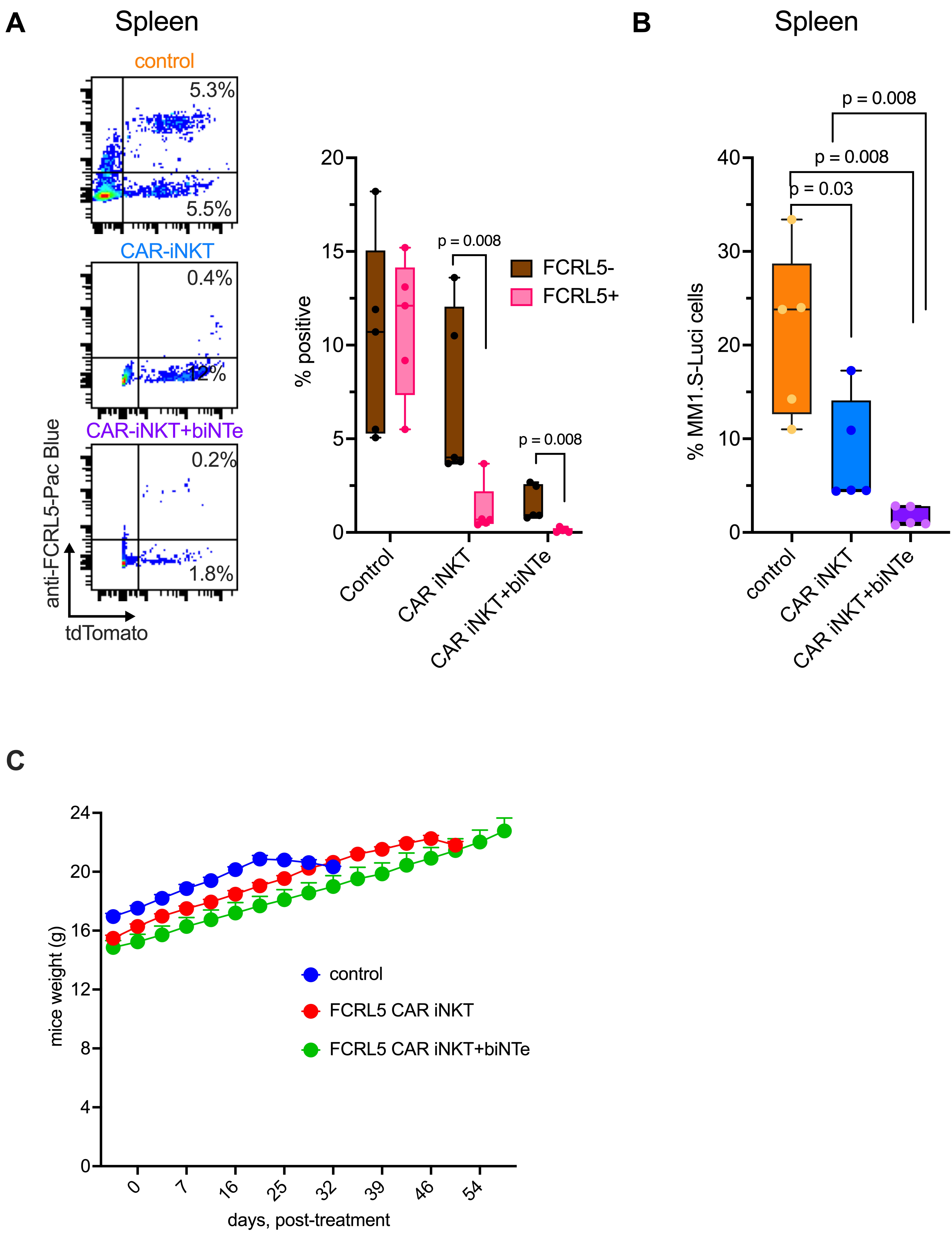
A
Spleen
control
5.3%
5.5%
CAR-iNKT
0.4%
12%
CAR-iNKT+biNTe
0.2%
1.8%
anti-FCRL5-Pac Blue
tdTomato
% positive
p = 0.008
p = 0.008
FCRL5-
FCRL5+
Control
CAR iNKT
CAR iNKT+biNTe
B
Spleen
p = 0.008
p = 0.008
p = 0.03
% MM1.S-Luci cells
control
CAR iNKT
CAR iNKT+biNTe
C
mice weight (g)
days, post-treatment
control
FCRL5 CAR iNKT
FCRL5 CAR iNKT+biNTe

Supplementary Figure 6 (related to Figure 6)

A. Flow cytometry analysis of frequency of MM1.S_FCRL5- and MM1.S-FCRL5+ cells in the spleen of treated and control mice at the end point shown as dot plot (left) and cumulative data for each group (right). (n=4-5 mice per group).

B. Flow cytometry analysis of total MM1.S myeloma burden as determined by tdTomato fluorescence marker in the spleen of treated and control mice at the end point. (n=4-5 mice per group).

C. Longitudinal weight measurement of animals from Figure 6B-H.

Two-tailed statistical analysis Mann-Whitney U test (A & B) performed. All error bars show Mean±SEM, and box and whiskers show minimum to maximum values with all data points.

All figure's error bars show Mean± SEM, and all the box and whiskers show minimum to maximum values with all the data points. $p <0.0001$ is referred as $p < 0.0001$. non significance measured by $p > 0.05$.
