# Supplementary material for: Enhancing iNKT cell immunotherapy through the integration of optimized CAR endodomains and iNKT engagers
Source: Nat Commun. 2026 Aug 14;17:9755. doi: 10.1038/s41467-026-76718-7 (PMC13572498; doi:10.1038/s41467-026-76718-7)
Supplement: Supplementary file 2 [file 41467_2026_76718_MOESM2_ESM.pdf]

Description of Additional Supplementary Files

Supplementary Dataset 1: The RNA sequencing data provided for NT vs CD28 group in sheet 1 (NT vs CD28z), NT vs 41BB group in sheet 2 (NT vs 41BBz) and comparison of CD28 and 41BB in sheet 3 (CD28z vs 41BBz). Common genes that are upregulated and downregulated in both groups are shown in sheet 4 (comparison_analysis).
