# Supplementary material for: Enhancing iNKT cell immunotherapy through the integration of optimized CAR endodomains and iNKT engagers
Source: Nat Commun. 2026 Aug 14;17:9755. doi: 10.1038/s41467-026-76718-7 (PMC13572498; doi:10.1038/s41467-026-76718-7)
Supplement: Supplementary file 4 [file 41467_2026_76718_MOESM5_ESM.pdf]

nature portfolio

Peer Review File

Enhancing the iNKT cell immunotherapy platform by combining optimised CAR endodomains with novel iNKT engagers

Corresponding Author: Dr Kanagaraju Ponnusamy

This file contains all reviewer reports in order by version, followed by all author rebuttals in order by version.

Version 0:

Reviewer comments:

Reviewer #1

(Remarks to the Author)
In this manuscript, Ponnusamy and colleagues tested multiple different CAR constructs and found that CD28-costimulated BCMA-CAR-iNKT cells had superior antitumor activity due to crosstalk between PLXND1 and SEMA4A on CAR-iNKT and MM cells, respectively. CAR-iNKT also outperformed CART in MM in part due to PLXND1 interactions. Then, the authors designed iNKT bispecific engagers. They found that iNKT + engagers led to improved tumor control, although still inferior to CAR-iNKT. Finally, authors showed that FCRL5 CAR-iNKT plus these engagers resulted in superior antitumor activity and reduced antigen escape through targeting FCRL5 and BCMA.

Innovation is moderate, as while the iNKT engager is novel, the optimized CAR endodomain is simply CD28z, and none of the combinations show curative potential in mice. Significance is moderate, as this strategy appears to be MM-specific rather than broadly applicable to a variety of cancer settings. Extensive clarification, discussion, and expansion of the existing data are needed to strengthen the authors' conclusions.

Major comments:
1. Authors should show BCMA antigen-specific proliferation of CAR-iNKT in Fig 1—Fig 1F-G show proliferation after IL15 and CD1d/a-Galcer, but BCMA-CAR stimulation would seem more relevant here.
2. Authors should offer additional explanation as to why PLXND1 was not found on CART cells. They say this was corroborated by scRNAseq data which showed low levels on memory T cells and absent on naïve T cells and Tregs (Supp Fig 2D), but much of the CART population would be effector T cells. Authors should comment on PLXND1 expression on TEM or TEMRA cells. Authors should also provide a reference for these published data.
3. Would overexpression of PLXDN1 on CART cells improve their antitumor activity in MM similar to that of CAR-iNKT cells?
4. Given the author's highlighting of significantly increased in vitro proliferation of CD28 CAR-iNKT cells, it is surprising that there was no significant difference in vivo shown in Fig 3H. Authors should assess serial peripheral blood samples to assess iNKT proliferation over time as well as in the bone marrow as shown. This applies to all other in vivo experiments shown as well.
5. The data in Fig 4 showing superiority of CAR-iNKT over CART are unconvincing. Tumor growth and survival appear to only differ by a few days between the groups. No statistics are shown in the tumor growth curve in Fig 4D, and there seems to be a lot of overlap between CART and CAR-iNKT curves. Authors should also assess if CAR-iNKT are superior to CART in CD1d-negative vs CD1d-positive models.
6. All in vivo models were performed with MM1S cells; additional cell lines, especially CD1d-positive cells (as Fig 1D shows MM1S is CD1d-negative), should be tested.
7. Authors briefly mention in the discussion that iNKT cells may be advantageous in MM due to targeting of CD1d-positive suppressive myeloid cells; this should be tested here as it may represent a major mechanism of antitumor activity in this context.
Minor comments:
1. For the cytotoxicity assay in Fig 1C, it is surprising that CD1d (on H929 but not MM1S) did not increase the toxicity against H929. Authors should discuss this observation.
2. Supp Fig 1D appears to show much lower IFNg production in the 3rd gen CARs compared to 2nd gen—authors should

discuss this.
3. Fig 1E and Supp Fig 1E should be merged into a single figure to compare all 5 CAR constructs together. The same is true for Fig 2D and Supp Fig 2A.
4. Significance is missing from several figures, e.g. Supp Fig 1K, Fig 2F, Fig 2H, Supp Fig 2E, Supp Fig 2L, Fig 3J, Fig 4D, Supp Fig 4A, Fig 5B, Fig 5D-F, Supp Fig 5J, Fig 6A, Fig 6D.
5. Significance and a combined tumor growth curve should be shown for Fig 3C in addition to the individual tumor growth curves.
6. Authors should put the specific days of the BLI plots shown in Fig 3B rather than “pre-treatment” and “post-treatment.” The same is true for Fig 4C, Fig 5K, and Fig 6C.
7. Supp Fig 3A appears to show significantly lower % MM1S cells in the spleen in CD28-OX40 compared to CD28. The representative flow plots to the left appear to have taken the lowest value (1%) for CD28 and highest value (7%) for CD28-OX40 based on the data points shown in the quantitative figure to the right. This is misleading.

Reviewer #2

(Remarks to the Author)
The manuscript entitled "Optimised iNKT-based, dual-targeting combinatorial therapy has enhanced anti-tumor activity against multiple myeloma" presents a comprehensive study positioning invariant Natural Killer T (iNKT) cells as a superior platform to conventional T cells for Chimeric Antigen Receptor (CAR) therapy in multiple myeloma. The authors systematically compare CAR signaling domains, identifying CD28z as superior in the iNKT context. They propose a novel mechanistic basis for this superiority involving the PLXND1-SEMA4A axis. Furthermore, the study introduces a novel "biNTe" (bispecific iNKT engager) and demonstrates that a combinatorial approach (FCRL5 CAR-iNKT + BCMA biNTe) can mitigate antigen escape. The study is highly relevant, addressing key challenges in anti-myeloma therapy: persistence, efficacy, and antigen escape. The flow from construct optimization to mechanistic discovery and finally to combinatorial application is logical and robust.

Major strengths of the paper include (1) the novel mechanism of avidity: The identification of the PLXND1-SEMA4A interaction as a driver of superior avidity in CD28z CAR-iNKT cells is a significant finding; (2) the platform comparison: the direct comparison between CAR-iNKT and CAR-T cells provides evidence of the superior potency of the iNKT platform in these specific models; (3) the dual-targeting strategy: the "biNTe" design allows for modular dual-targeting. Demonstrating that FCRL5 CAR + BCMA biNTe can control heterogeneous tumors (containing antigen-negative subclones) is a crucial proof-of-concept for preventing relapse; (4) the use of primary tissue: the inclusion of primary bone marrow samples from myeloma patients to test specificity strengthens the translational relevance of the findings.

Major comments:
1. The manuscript claims CD28z is superior to 4-1BBz in iNKT cells, contrasting with the general consensus in CAR-T literature where 4-1BB promotes better persistence. While "short-term proliferation" and "long-term expansion in presence of IL-15" are mentioned. Did the authors perform a repetitive stress test (serial rechallenge) without exogenous cytokines to mimic the tumor microenvironment more closely? Were exhaustion markers (PD-1, TIM-3, LAG-3) quantified on the CD28z vs. 4-1BBz iNKT cells after long-term in vivo exposure?

2. Regarding the PLXND1-SEMA4A Axis:
- While CRISPR knockout of SEMA4A reduced avidity, did the authors attempt to overexpress PLXND1 in the 4-1BBz CAR-iNKT cells? Demonstrating that 4-1BBz cells gain CD28z-like avidity through PLXND1 overexpression would prove sufficiency.
- The text mentions "additional work will be required to determine signaling pathways." While acceptable, a brief investigation into whether PLXND1 signaling converges with TCR or CAR signaling (e.g., phosphorylation of downstream targets) would significantly strengthen the mechanistic claim.

3. Regarding safety and toxicity; the authors mention reduced risk of CRS/neurotoxicity as a general benefit of iNKTs. However, in the dual-targeting experiments (CAR + biNTe), was there any evidence of off-tumor toxicity or cytokine storms in the mouse models? The systemic administration of a bispecific engager alongside a potent CAR warrants specific safety data.

4. The study uses NSG mice, which lack a functional adaptive immune system. Given that a key benefit of iNKT cells is "bridging innate and adaptive immunity" (trans-activation of NKs, DCs, and T cells), the current model likely underestimates the full therapeutic potential. The authors should discuss this limitation more explicitly, or if possible, reference any immunocompetent models.

Minor comments:
5. In the Introduction (Line 79), the text reads "protect from acute gram-versus-host disease." This should be corrected to "graft-versus-host disease."

6. Clarification on biNTe Dosing: In the in vivo methods, the specific rationale for the biNTe dosing schedule (twice a week) versus the half-life of the construct should be briefly clarified.

7. Ensure all flow cytometry plots in the supplementary figures clearly distinguish between donor variability vs. construct

variability.

Reviewer #3

(Remarks to the Author)
This exhaustive manuscript is based on prior extensive work on CAR-iNKT by this group, which have long been leaders in the roles and therapeutic potential of iNKT cells, particularly in Hematological diseases including malignancies, as well as several others groups' work (with a greater emphasis on solid cancers, though also for example, CD19 CAR-iNKT, as cited, confirming conclusions to date across the field).
This new study takes all this much further and in different directions, with 5 different ICDs tested in vitro and in vivo, identifying an optimal ICD (not obvious, more signaling domains is not better !), a new parallel receptor - ligand interaction helping CAR-INKT be better than conventional CAR-T in this context and also preparing and evaluating several iNKT cell engagers, as another way of helping iNKT find the tumor cells.
CAR ICD evaluation: multiple donor iNKT were used to compare ICDs with the same BCMA CAR ICD widely used in the field to ensure results generalise. CD28z CAR-iNKT were significantly more proliferative and higher cell:cell avidity, though similar in vitro killing with multiple targets.
Interestingly, the difference was reflected in another protein-protein interaction than the CAR or iTCR. Differential RNA suggested candidates between different ICD: CD28z CAR vs. 4-1BBz CAR. Checking other less active ICDs confirmed general result that PLXND1 was the biggest difference, missing on CAR-T even with CD28z CAR. @ PLXND1 reduced only CD28z CAR-iNKT avidity. Only the SEMA4A known ligand for PLXND1 was consistently expressed by Myeloma lines. KO of SEMA4A reduced CD28z CAR avidity to 4-1BBz CAR levels (which were not affected by KO of SEMA4A), thus formally identifying the key interaction. Since using a different ECD didn't change the differences in CD28z CAR vs. 4-1BBz CAR-iNKT proliferation and induction of PLXND1, apparently signaling via the CD28z ICD in iNKT specifically induces a new receptor for another myeloma target protein not induced by the same CAR in T cells, a remarkable finding.
In vivo modelling with conditions to pick up differences in activity suggested that CD28z CAR-iNKT was the most potent in delaying tumor growth and need to sacrifice the mice amongst the five CAR types. In 3C, was CD28 statistically different from 4-1BB or CD28-OX40 (also next best in 3E) ? The shape of the curves only at last time point suggests benefit over those, even if not, so description could be modified for precision. 3D confirms a benefit.
@PLXND1 reduced that protection significantly. CD28z CAR-iNKT looked also more potent than CD28z CAR-T in vitro and in vivo, supporting the additional benefit of the PLXND1 interaction and other iNKT tumor ligands (DNAM-1, NKG2D). However, 4D may not be statistical either. Please address description if so, the benefit of iNKT formally supported either side.
Finally, the group made 2 'biNTe' using the well-established @Vb11 mAb C21 as 'proof-of-principle'. Vb11 is commonly (though not essentially) found on iNKT, in the hope this would not inhibit iTCR-CD1d interactions (unlike direct @Va24Ja18 mAb would be more likely to do). An established @BCMA mAb ScFv was the other end of the 2 versions of engager made. Both bound specifically to iNKT and BCMA and killed only BCMA+ myeloma calls with iNKT, but not T cells (generic T cells, presumably containing too few iNKT to be measurably active) or BCMA-negative myeloma line. aGalcer enhancement still took place, confirming @Vb11 did not block iTCR binding. Interestingly, even monovalent Ab had activity (5D). Please comment accordingly. Importantly, also killed were primary myeloma cells greater than normal marrow cells from 5 patients (spontaneous death is higher with primary cells, of course, so a higher background). Killing in vitro and in vivo anti-tumor activity of this first biNTe + iNKT were positive, but less than CAR-iNKT. In combination, other myeloma target FcRL5 CAR-iNKT alone or + BCMA biNTe were used with mixtures of myeloma lines where BCMA and FcRL5 are expressed by 100% and 50%, respectively. When both therapies were present, disease progressed slower with more FcRL5+ cells killed. BCMA biNTe reduced total myeloma cells and prolonged survival in vivo, even in the mixed tumor mice.

As discussion points:
• might the same optimised CAR &/or appropriate other engager work well in other innate killer cells ?
• what about CD28 signaling might induce PLXND1 specifically.

Minor point: consistency of FcRL5 vs. FCRL5.

Version 1:

Reviewer comments:

Reviewer #1

(Remarks to the Author)
The authors have adequately addressed the questions and concerns.

Reviewer #2

(Remarks to the Author)
None

Reviewer #3

(Remarks to the Author)

The Authors have completed a major revision of the already substantial manuscript, incorporating new data as well as many edits as suggested by the 3 reviewers. The MS is now an important and thorough contribution, I have no further comments or issues to raise.

Open Access This Peer Review File is licensed under a Creative Commons Attribution 4.0 International License, which permits use, sharing, adaptation, distribution and reproduction in any medium or format, as long as you give appropriate credit to the original author(s) and the source, provide a link to the Creative Commons license, and indicate if changes were made.
In cases where reviewers are anonymous, credit should be given to 'Anonymous Referee' and the source.
The images or other third party material in this Peer Review File are included in the article's Creative Commons license, unless indicated otherwise in a credit line to the material. If material is not included in the article's Creative Commons license and your intended use is not permitted by statutory regulation or exceeds the permitted use, you will need to obtain permission directly from the copyright holder.
To view a copy of this license, visit https://creativecommons.org/licenses/by/4.0/

Response to the reviewers

Article title: Enhancing the iNKT cell immunotherapy platform by combining optimised CAR endodomains with novel iNKT engagers.

REVIEWER COMMENTS

Reviewer #1 (Remarks to the Author):

In this manuscript, Ponnusamy and colleagues tested multiple different CAR constructs and found that CD28-costimulated BCMA-CAR-iNKT cells had superior antitumor activity due to crosstalk between PLXND1 and SEMA4A on CAR-iNKT and MM cells, respectively. CAR-iNKT also outperformed CART in MM in part due to PLXND1 interactions. Then, the authors designed iNKT bispecific engagers. They found that iNKT + engagers led to improved tumor control, although still inferior to CAR-iNKT. Finally, authors showed that FCRL5 CAR-iNKT plus these engagers resulted in superior antitumor activity and reduced antigen escape through targeting FCRL5 and BCMA.

Innovation is moderate, as while the iNKT engager is novel, the optimized CAR endodomain is simply CD28z, and none of the combinations show curative potential in mice. Significance is moderate, as this strategy appears to be MM-specific rather than broadly applicable to a variety of cancer settings. Extensive clarification, discussion, and expansion of the existing data are needed to strengthen the authors' conclusions.

We thank the reviewer for the constructive appraisal of our work. We feel that in the context of the two clinically approved BCMA CAR-T products ciltacel and idecel having a 4-1BBz endo-domain, identification of CD28z as the optimal endo-domain for CAR-iNKT immunotherapy is novel; more so is our identification and validation of the novel PLXND1-SEMA4A interaction as the mechanism that underpins the superior anti-myeloma effect of CD28z CAR-iNKT. We also note that our myeloma model is considerably more aggressive than previously published models involving the same MM1.S myeloma cell line. Instead of the reported 0.5-1x10e6 cells (for example PMID: 34841295 and PMID: 38744947), in our model we used 5-7x10e^{6} myeloma cells.

Major comments:

1. Authors should show BCMA antigen-specific proliferation of CAR-iNKT in Fig 1—Fig 1F-G show proliferation after IL15 and CD1d/a-Galcer, but BCMA-CAR stimulation would seem more relevant here.

The presented data does not explore proliferation per se but expansion under the described manufacturing protocol. To address the reviewer's comment, we performed cell proliferation assay after exposing CAR iNKT cells with

different co-stimulatory molecules to the target MM1.S cells at 1:1 E:T ratio. We find that CD28z induces more proliferation of BCMA CAR iNKT cells as compared to 41BBz and other co-stimulatory molecules after CAR-mediated stimulation (Figure 1H and Suppl Fig 1E, and lines 132-138 and 578-590 highlighted).

2. Authors should offer additional explanation as to why PLXND1 was not found on CART cells. They say this was corroborated by scRNAseq data which showed low levels on memory T cells and absent on naïve T cells and Tregs (Supp Fig 2D), but much of the CART population would be effector T cells. Authors should comment on PLXND1 expression on TEM or TEMRA cells. Authors should also provide a reference for these published data.

We obtained scRNA seq data from the Human protein Atlas, originally reported by Schmiedel et al PMID: 30449622) and Monaco et al (PMID: 30726743; now also shown in Supp Fig 2D). In the Monaco et al dataset amongst CD4+ and CD8+ T cell subsets, CD4+ TEMRA express the highest PLXND1. However, since CD4+ TEMRA are only as small fraction of all T cells (0.3-<18%; PMID: 28069807), they wouldn't be expected to have an appreciable effect on expression of PLXND1 when total T cell are assessed by flow-cytometry. Similarly since frequency of CD8+ is only high in advanced age, it is likely that in our young donors we didn't have substantial numbers of such cells.

We clarify the above in the Results and Methods (Data analysis and software), where the two original relevant references are also cited (text line 174-176 and 741-743 and 869-873).

3. Would overexpression of PLXDN1 on CART cells improve their antitumor activity in MM similar to that of CAR-iNKT cells?

To address this question, we cloned the 5.8kb PLXND1 cDNA into a lentiviral, bicistronic vector that also encodes GFP and transduced the PLXND1-GFP or GFP-only lentivirus into effector cells. After flow-sorting of GFP/PLXND1-expressing cells, we obtained highly pure CAR-T and CAR-iNKT effectors which we used to perform avidity assays. The results show that over-expression of PLXND1 in CAR-T cells or in 4-1BBz CAR-iNKT enhances their avidity to the same level as that of CD28z CAR-iNKT cells. These data further support the notion that higher PLXND1 expression underpins the higher avidity of CD28z CARs when introduced into iNKT and eventually the superior anti-myeloma activity of CD28z CAR-iNKT when compared to 4-1BBz CAR-iNKT.

These results are discussed in lines 210-217 and 571-576 and are shown in Figure 2I and Suppl Fig. 2M of the revised manuscript.

4. Given the author's highlighting of significantly increased in vitro proliferation of CD28 CAR-iNKT cells, it is surprising that there was no significant difference in vivo

shown in Fig 3H. Authors should assess serial peripheral blood samples to assess iNKT proliferation over time as well as in the bone marrow as shown. This applies to all other in vivo experiments shown as well.

Higher ex vivo expansion during manufacturing does not necessarily mean higher proliferation in vivo. However, the in vivo data is still of importance, because in the CAR-T context it has been shown that 4-1BBz CAR-T persist longer than CD28z counterparts; here we show that in the context of CAR-iNKT immunotherapy, persistence of CD28z CAR-iNKT is at least the same as of that of 4-11Bz CAR-iNKT.

5. The data in Fig 4 showing superiority of CAR-iNKT over CART are unconvincing. Tumor growth and survival appear to only differ by a few days between the groups. No statistics are shown in the tumor growth curve in Fig 4D, and there seems to be a lot of overlap between CART and CAR-iNKT curves.

This is a stress test where the potency of limiting, subtherapeutic number of effectors is compared. It is therefore to be expected that difference in survival would be short; nevertheless, these are highly significant.

There is moderate difference in the BLI signal because as well as medullary and splenic disease, in this model, there is also extramedullary disease. However, when tumour burden is more directly and granularly assessed in the BM and spleen the differences are highly significant demonstrating higher efficacy of CAR-iNKT over CAR-T.

Please note that in our MM1.S model we transfer x5 times more tumor cells compared to other reports, for example PMID: 34841295 and PMID: 38744947. This is in order to shorten the natural history of the disease and thus avoid aGVHD by CAR-T which has been observed in other reports in which only 10e6 MM1.S were used. This allows direct comparison of CAR-iNKT vs CAR-T without aGVHD becoming a confounder. The downside is a more aggressive model of myeloma that includes extramedullary disease.

In addition, we have performed comparison of BCMA CAR-T and CAR iNKT efficacy in vivo in another sbc myeloma model using the CD1d+BCMA+ H929 cells. Treatment with subtherapeutic dose 1x10e6 effectors shows a superior tumor control by CD28z BCMA CAR iNKT cells as compared to T cell counterparts. This new data is shown in Figure 4H-J and Suppl Fig 4D and discussed in text lines 260-267 and 607-610.

Authors should also assess if CAR-iNKT are superior to CART in CD1d-negative vs CD1d-positive models.

We have now performed in vivo assays using the CD1d+ H929 as well as the CD1d- MM1.S myeloma cells. In both models CAR-iNKT outperform CAR-T (revised Figure 4). This new data is shown in Figure 4H-J and Suppl Fig. 4D and discussed in text lines 260-267 and 607-610.

6. All in vivo models were performed with MM1S cells; additional cell lines, especially CD1d-positive cells (as Fig 1D shows MM1S is CD1d-negative), should be tested.

See response to point 5.

7. Authors briefly mention in the discussion that iNKT cells may be advantageous in MM due to targeting of CD1d-positive suppressive myeloid cells; this should be tested here as it may represent a major mechanism of antitumor activity in this context.
This mechanism of anti-tumor activity of iNKT cell-based immunotherapy has already been extensively shown by others and are referenced in our manuscript (refs 10,15,16,42).

Minor comments:
1. For the cytotoxicity assay in Fig 1C, it is surprising that CD1d (on H929 but not MM1S) did not increase the toxicity against H929. Authors should discuss this observation.

This is not surprising because different cell lines have different levels of sensitivity to the cytotoxic effect of the same effectors. Therefore, this cannot be the direct comparation for CD1d function.

2. Supp Fig 1D appears to show much lower IFNg production in the 3rd gen CARs compared to 2nd gen—authors should discuss this.

As we also discuss in the manuscript, there are no statistically significant differences in GZMB and IFNgamma production amongst the 2nd and 3rd gen CAR-iNKT. In the revised figures we indicate this lack of significant differences by 'ns'.

3. Fig 1E and Supp Fig 1E should be merged into a single figure to compare all 5 CAR constructs together. The same is true for Fig 2D and Supp Fig 2A.

Fig 1E and supp Fig 1E merged into Fig 1E and the text has been altered accordingly.

Fig 2D and Supp Fig 2A has been merged into Fig 2D and the text has been altered accordingly.

4. Significance is missing from several figures, e.g. Supp Fig 1K, Fig 2F, Fig 2H, Supp Fig 2E, Supp Fig 2L, Fig 3J, Fig 4D, Fig 5B, Fig 5D-F, Supp Fig 5J, Fig 6A, Fig 6D.

Where appropriate we added missing significance values to relevant graphs.

5. Significance and a combined tumor growth curve should be shown for Fig 3C in addition to the individual tumor growth curves.

This additional graph has now been added as Suppl Fig 3A. CD28z CAR-iNKT-treated animals had significantly lower BLI signal than animals treated with the other four CAR-iNKT.

6. Authors should put the specific days of the BLI plots shown in Fig 3B rather than “pre-treatment” and “post-treatment.” The same is true for Fig 4C, Fig 5K, and Fig 6C.

Changes made as suggested.

7. Supp Fig 3A appears to show significantly lower % MM1S cells in the spleen in CD28-OX40 compared to CD28. The representative flow plots to the left appear to have taken the lowest value (1%) for CD28 and highest value (7%) for CD28-OX40 based on the data points shown in the quantitative figure to the right. This is misleading.

We have replaced these flow-cytometry plots with more representative examples.

Reviewer #2 (Remarks to the Author):

The manuscript entitled "Optimised iNKT-based, dual-targeting combinatorial therapy has enhanced anti-tumor activity against multiple myeloma" presents a comprehensive study positioning invariant Natural Killer T (iNKT) cells as a superior platform to conventional T cells for Chimeric Antigen Receptor (CAR) therapy in multiple myeloma. The authors systematically compare CAR signaling domains, identifying CD28z as superior in the iNKT context. They propose a novel mechanistic basis for this superiority involving the PLXND1-SEMA4A axis. Furthermore, the study introduces a novel "biNTe" (bispecific iNKT engager) and demonstrates that a combinatorial approach (FCRL5 CAR-iNKT + BCMA biNTe) can mitigate antigen escape. The study is highly relevant, addressing key challenges in anti-myeloma therapy: persistence, efficacy, and antigen escape. The flow from construct optimization to mechanistic discovery and finally to combinatorial application is logical and robust.

Major strengths of the paper include (1) the novel mechanism of avidity: The identification of the PLXND1-SEMA4A interaction as a driver of superior avidity in CD28z CAR-iNKT cells is a significant finding; (2) the platform comparison: the direct comparison between CAR-iNKT and CAR-T cells provides evidence of the superior potency of the iNKT platform in these specific models; (3) the dual-targeting strategy: the "biNTe" design allows for modular dual-targeting. Demonstrating that FCRL5 CAR + BCMA biNTe can control heterogeneous tumors (containing antigen-negative subclones) is a crucial proof-of-concept for preventing relapse; (4) the use of primary

tissue: the inclusion of primary bone marrow samples from myeloma patients to test specificity strengthens the translational relevance of the findings.

We thank the reviewer for the overall positive appraisal of our work and their constructive comments

Major comments:

1. The manuscript claims CD28z is superior to 4-1BBz in iNKT cells, contrasting with the general consensus in CAR-T literature where 4-1BB promotes better persistence. While "short-term proliferation" and "long-term expansion in presence of IL-15" are mentioned. Did the authors perform a repetitive stress test (serial rechallenge) without exogenous cytokines to mimic the tumor microenvironment more closely? Were exhaustion markers (PD-1, TIM-3, LAG-3) quantified on the CD28z vs. 4-1BBz iNKT cells after long-term in vivo exposure?

In response to the reviewer's comments we performed tumor rechallenge assay, in which BCMA-expressing MM1.S cells were added to the same pool of either CD28z or 41BBz anti-BCMA CAR-iNKT without exogenous cytokine. In this longitudinal assay, cytotoxicity against myeloma were monitored at 1:1 E:T ratio for up to 4 challenges in the interval of 4 days concomitantly by real time Incucyte imaging. The exhaustion markers PD-1, LAG-3, TIM-3 were measured before co-culture with target cells MM1.S and after 4 cycles of tumor re-challenge.

We did not find significant differences in the cytotoxic activity of CD28z CAR-iNKT as compared to 41BBz CAR iNKT cells or expression of exhaustion markers PD-1, LAG-3, TIM-3 (Fig 1I and Suppl Fig 1K-M). These data suggest that superior efficacy of CD28z CAR iNKT cells *in vivo* reflects enhanced avidity of CD28z cells rather than cell exhaustion.

These results are discussed in text line 141-145 and 578-590 and are shown in Figure 1I and Suppl Fig. 1K-M of the revised manuscript.

2. Regarding the PLXND1-SEMA4A Axis:

- While CRISPR knockout of SEMA4A reduced avidity, did the authors attempt to overexpress PLXND1 in the 4-1BBz CAR-iNKT cells? Demonstrating that 4-1BBz cells gain CD28z-like avidity through PLXND1 overexpression would prove sufficiency.

To address this question, we cloned the 5.8kb PLXND1 cDNA into a lentiviral bicistronic vector that also encodes GFP and transduced the PLXND1-GFP or GFP-only lentivirus into effector cells. After flow-sorting of GFP/PLXND1-expressing cells, we obtained highly pure CAR-T and CAR-iNKT effectors which we used to perform avidity assays. The results show that over-expression of PLXND1 in CAR-T cells or in 4-1BBz CAR-iNKT enhances their avidity to the same or higher level as that of CD28z CAR-iNKT cells. These data further support the notion that higher PLXND1 expression underpins the

higher avidity of CD28z CARs when introduced into iNKT cells and eventually the superior anti-myeloma activity of CD28z CAR-iNKT when compared to 4-1BBz counterparts.

These results are discussed in lines 210-217 and 571-576 and are shown in Figure 2I and Suppl Fig. 2M of the revised manuscript.

- The text mentions "additional work will be required to determine signaling pathways." While acceptable, a brief investigation into whether PLXND1 signaling converges with TCR or CAR signaling (e.g., phosphorylation of downstream targets) would significantly strengthen the mechanistic claim.

We thank of the reviewer for the suggestion; studying signalling pathways properly would require a whole new project which we intend to pursue in the future.

3. Regarding safety and toxicity; the authors mention reduced risk of CRS/neurotoxicity as a general benefit of iNKTs. However, in the dual-targeting experiments (CAR + biNTe), was there any evidence of off-tumor toxicity or cytokine storms in the mouse models? The systemic administration of a bispecific engager alongside a potent CAR warrants specific safety data.

We observed no clinical evidence of off-tumor or CRS toxicity imparted by the biNTe alone or in combination with CAR-iNKT and the weight of the corresponding mice over time was almost identical (included in the revised man in Suppl Fig 4C&D & 5J&M).

With regards safety of CAR-iNKT, early clinical studies of unmodified or CAR-iNKT demonstrate remarkable lack of CRS, ICANS or alloreactivity (see ref 18 and PMID: 38321023)

We discuss this in the sections of Results for Figures 4 & 5 and show the weight data in Suppl Fig 4B&C & 5J&M.

4. The study uses NSG mice, which lack a functional adaptive immune system. Given that a key benefit of iNKT cells is "bridging innate and adaptive immunity" (trans-activation of NKs, DCs, and T cells), the current model likely underestimates the full therapeutic potential. The authors should discuss this limitation more explicitly, or if possible, reference any immunocompetent models.

We agree that our NSG model likely underestimates the full therapeutic potential of CAR-iNKT. We had already briefly discussed and referenced immunocompetent models, see Discussion and refs [10,15,16,42] cited there.

Minor comments:
5. In the Introduction (Line 79), the text reads "protect from acute gram-versus-host disease." This should be corrected to "graft-versus-host disease."

Thank you for highlighting this typo, it is now corrected

6. Clarification on biNTe Dosing: In the in vivo methods, the specific rationale for the biNTe dosing schedule (twice a week) versus the half-life of the construct should be briefly clarified.
We did not perform formal pharmacokinetic studies. The level and schedule of the dosing were based on the dosing scheme of T cell-directed bispecific engagers in pre-clinical models of myeloma, for example the CD3XBCMA TCE Teclistamab (PMID: 32956453).

7. Ensure all flow cytometry plots in the supplementary figures clearly distinguish between donor variability vs. construct variability.
This has been addressed

Reviewer #3 (Remarks to the Author):

This exhaustive manuscript is based on prior extensive work on CAR-iNKT by this group, which have long been leaders in the roles and therapeutic potential of iNKT cells, particularly in Hematological diseases including malignancies, as well as several others groups' work (with a greater emphasis on solid cancers, though also for example, CD19 CAR-iNKT, as cited, confirming conclusions to date across the field).
This new study takes all this much further and in different directions, with 5 different ICDs tested in vitro and in vivo, identifying an optimal ICD (not obvious, more signaling domains is not better !), a new parallel receptor - ligand interaction helping CAR-INKT be better than conventional CAR-T in this context and also preparing and evaluating several iNKT cell engagers, as another way of helping iNKT find the tumor cells.
CAR ICD evaluation: multiple donor iNKT were used to compare ICDs with the same BCMA CAR ICD widely used in the field to ensure results generalise. CD28z CAR-iNKT were significantly more proliferative and higher cell:cell avidity, though similar in vitro killing with multiple targets.
Interestingly, the difference was reflected in another protein-protein interaction than the CAR or iTCR. Differential RNA suggested candidates between different ICD: CD28z CAR vs. 4-1BBz CAR. Checking other less active ICDs confirmed general result that PLXND1 was the biggest difference, missing on CAR-T even with CD28z CAR. @ PLXND1 reduced only CD28z CAR-iNKT avidity. Only the SEMA4A known ligand for PLXND1 was consistently expressed by Myeloma lines. KO of SEMA4A reduced CD28z CAR avidity to 4-1BBz CAR levels (which were not affected by KO of SEMA4A), thus formally identifying the key interaction. Since using a different ECD didn't change the differences in CD28z CAR vs. 4-1BBz CAR-iNKT proliferation and induction of PLXND1, apparently signaling via the CD28z ICD in iNKT specifically induces a new receptor for another myeloma target protein not induced by the same CAR in T cells, a remarkable finding.

In vivo modelling with conditions to pick up differences in activity suggested that CD28z CAR-iNKT was the most potent in delaying tumor growth and need to sacrifice the mice amongst the five CAR types.

We thank the reviewer for the overall positive appraisal of our work and their constructive comments.

In 3C, was CD28 statistically different from 4-1BB or CD28-OX40 (also next best in 3E) ? The shape of the curves only at last time point suggests benefit over those, even if not, so description could be modified for precision. 3D confirms a benefit.

We added combined graph in Suppl Fig. 3A which summarises and compares the BLI signal from all ICD. As indicated on the panel, statistical analysis shows, compared to other ICD, significant reduction of the BLI signal in CD28z CAR-iNKT-treated animals.

@PLXND1 reduced that protection significantly. CD28z CAR-iNKT looked also more potent than CD28z CAR-T in vitro and in vivo, supporting the additional benefit of the PLXND1 interaction and other iNKT tumor ligands (DNAM-1, NKG2D).

However, 4D may not be statistical either. Please address description if so, the benefit of iNKT formally supported either side.

In Figure 4D, although BLI signal differences were not significant between CAR-T and CAR-iNKT, myeloma cells enumerated in the bone marrow and spleens were significantly fewer in 28zCAR-iNKT treated animals while survival of CD28z CAR-iNKT-treated animals was also clearly significantly longer than that of CAR-T treated ones (Fig 4G). Please note that in systemic MM1.S myeloma mouse model, the disease is not restricted haematopoietic organs bone marrow and spleen but they also develop extramedullary disease.

Furthermore, in an additional in vivo systemic model, 5x10e6 H929 myeloma cells mixed with Matrigel, were injected subcutaneously into the flank of NSG mice and when tumors became measurable, mice received the limiting number of 1x10e6 CD28z or 4-1BB anti-BCMA CAR-iNKT cells intravenously. We found that CD28z CAR-iNKT were significantly more potent in controlling tumor growth as compared to untreated and 4-1BB CAR-iNKT-treated mice.

This new data shown in Figure 4H-J and discussed in text lines 260-267 and 607-610, further consolidate the higher efficacy of CAR-iNKT over CAR-T in pre-clinical myeloma as we and others also have demonstrated for B cell lymphoma and high risk acute lymphoblastic leukemia.

Finally, the group made 2 ‘biNTe’ using the well-established @Vb11 mAb C21 as ‘proof-of-principle’. Vb11 is commonly (though not essentially) found on iNKT, in the hope this would not inhibit iTCR-CD1d interactions (unlike direct @Va24Ja18 mAb would be more likely to do). An established @BCMA mAb ScFv was the other end of the 2 versions of engager made. Both bound specifically to iNKT and BCMA and killed only BCMA+ myeloma calls with iNKT, but not T cells (generic T cells, presumably containing too few iNKT to be measurably active) or BCMA-negative

myeloma line. aGalcer enhancement still took place, confirming @Vb11 did not block iTCR binding. Interestingly, even monovalent Ab had activity (5D). Please comment accordingly.

We added a brief comment in Discussion with regards the different potency of mono vs bivalent engager likely reflecting differences in affinity and avidity.

Importantly, also killed were primary myeloma cells greater than normal marrow cells from 5 patients (spontaneous death is higher with primary cells, of course, so a higher background). Killing in vitro and in vivo anti-tumor activity of this first biNTe + iNKT were positive, but less than CAR-iNKT. In combination, other myeloma target FcRL5 CAR-iNKT alone or + BCMA biNTe were used with mixtures of myeloma lines where BCMA and FcRL5 are expressed by 100% and 50%, respectively. When both therapies were present, disease progressed slower with more FcRL5+ cells killed. BCMA biNTe reduced total myeloma cells and prolonged survival in vivo, even in the mixed tumor mice.

As discussion points:

• might the same optimised CAR &/or appropriate other engager work well in other innate killer cells ?

This is an interesting point, to our knowledge there is no systematic comparison of ICDs in CAR-NK and gdT cells. We mention this in Discussion of the revised manuscript

• what about CD28 signaling might induce PLXND1 specifically.

It would be very interesting to address thus question in future work.

Minor point: consistency of FcRL5 vs. FCRL5.

We adopted FCRL5 across the board.
